# Carbon Nanodots with Nearly Unity Fluorescent Efficiency Realized via Localized Excitons

**DOI:** 10.1002/advs.202203622

**Published:** 2022-08-24

**Authors:** Qing Lou, Qingchao Ni, Chunyao Niu, Jianyong Wei, Zhuangfei Zhang, Weixia Shen, Chenglong Shen, Chaochao Qin, Guangsong Zheng, Kaikai Liu, Jinhao Zang, Lin Dong, Chong‐Xin Shan

**Affiliations:** ^1^ Henan Key Laboratory of Diamond Optoelectronic Materials and Devices Key Laboratory of Materials Physics Ministry of Education, and School of Physics and Microelectronics Zhengzhou University Zhengzhou 450052 China; ^2^ State Key Laboratory of Advanced Optical Communication Systems and Networks University of Michigan–Shanghai Jiao Tong University Joint Institute Shanghai Jiao Tong University Shanghai 200240 China; ^3^ Henan Key Laboratory of Infrared Materials and Spectrum Measures and Applications College of Physics and Materials Science Henan Normal University Xinxiang 453007 China

**Keywords:** carbon nanodot, light‐emitting diode, localized exciton, symmetry breaking

## Abstract

Carbon nanodots (CDs) have emerged as an alternative option for traditional nanocrystals due to their excellent optical properties and low toxicity. Nevertheless, high emission efficiency is a long‐lasting pursuit for CDs. Herein, CDs with near‐unity emission efficiency are prepared via atomic condensation of doped pyrrolic nitrogen, which can highly localize the excited states thus lead to the formation of bound excitons and the symmetry break of the *π*–electron conjugation. The short radiative lifetimes (<8 ns) and diffusion lengths (<50 nm) of the CDs imply that excitons can be efficiently localized by radiative recombination centers for a defect‐insensitive emission of CDs. By incorporating the CDs into polystyrene, flexible light‐converting films with a high solid‐state quantum efficiency of 84% and good resistance to water, heating, and UV light are obtained. With the CD–polymer films as light conversion layers, CD‐based white light‐emitting diodes (WLEDs) with a luminous efficiency of 140 lm W^−1^ and a flat‐panel illumination system with lighting sizes of more than 100 cm^2^ are achieved, matching state‐of‐the‐art nanocrystal‐based LEDs. These results pave the way toward carbon‐based luminescent materials for solid‐state lighting technology.

## Graphical abstract

Carbon nanodots (CDs) with near‐unity emission efficiency are prepared via atomic condensation of doped pyrrolic nitrogen, which can highly localize the excited states thus lead to the formation of bound excitons and the symmetry break of the *π*–electron conjugation. These results pave the way toward carbon‐based luminescent materials for solid‐state lighting technology.

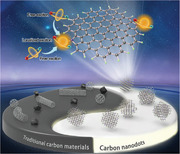

## 1 Introduction

Fluorescent carbon nanodots (CDs), astonishingly shining from “totally black” carbon‐based materials, have attracted extensive attention in recent years for their excellent luminescent properties, low cost, and high biocompatibility.^[^ ^1^, ^2^, ^3^, ^4^, ^5^ ^]^ Various potential applications including optical sensing, bioimaging, photo(electro)catalysis, and optoelectronic devices have been exploited from CDs.^[^ ^6^, ^7^, ^8^, ^9^, ^10^, ^11^, ^12^, ^13^, ^14^, ^15^, ^16^ ^]^ Since the first report of this material about a decade ago,^[^ ^17^ ^]^ great progress in optical emission of the CDs has been achieved with the advance of multifarious synthetic procedures such as laser ablation,^[^ ^17^ ^]^ electrochemical oxidation,^[^ ^18^ ^]^ solvothermal strategy,^[^ ^19^, ^20^ ^]^ and microwave‐assisted pyrolysis.^[^ ^21^, ^22^ ^]^ Nevertheless, the unique structure of CDs built on the graphite‐type sp^2^ and diamond‐type or disordered sp^3^ hybridized carbon frameworks raises the barrier for insight into the emission processes and advance in the luminescent performance and applications. Hence, continuous efforts are dedicated to develop CDs with high emission efficiency and controllable reabsorption for their luminescence‐related applications, especially in cost‐effective solid‐state lighting sources.

Developing high‐performance solid‐state lighting devices is an ideal way to save energy and alleviate environmental pollution for the social sustainable development. White light‐emitting diodes (WLEDs) appear to be the most promising alternative technologies for solid‐state lighting due to their high efficiency, long lifetime, environmental friendliness, etc.^[^ ^23^, ^24^ ^]^ Nowadays, most commercial and energy‐efficient WLEDs are achieved by combining the blue LED chips with yellow‐emitting rare‐earth‐based phosphors owing to their excellent theoretical luminous efficiency (LE) and chromatic stability. Nevertheless, those inevitable issues, for instance, extremely low abundance in the Earth's crust, nonrenewability, and enormous environmental pressures produced in the process of rare‐earth mining, refining, recovery, and application severely hinder the fully application of rare‐earth‐based phosphors.^[^ ^25^ ^]^ One appealing solution for high‐performance WLEDs is by utilization of novel semiconductor nanocrystals like CDs. Without inherent challenges caused by the noble and toxic metal elements, or the unsatisfactory stability for the traditional colloidal quantum dots (QDs)^[^ ^23^, ^26^ ^]^ and emerging perovskite QD materials,^[^ ^27^, ^28^ ^]^ CDs excel recently as a family of important candidate phosphors for the low‐cost and nonpolluting WLEDs.^[^ ^29^, ^30^, ^31^, ^32^, ^33^ ^]^ Meanwhile, mass production of CDs could be achieved from abundant and renewable carbon sources including organic molecules and natural reservoirs like vegetables, fruits, plants, and even meat.^[^ ^13^, ^34^ ^]^ However, CD‐based phosphors still suffer from low LE, mainly due to their relatively low photoluminescence quantum yield (PLQY) and drastic optical losses induced by the reabsorption in the luminescence conversion layer. Hence, further development of a carbon‐based luminescent system is essential for achieving efficient WLED with CD phosphors.

An important strategy to achieve high‐efficiency luminescence is to form localized excitons via immobilizing the photogenerated electron–hole pairs to the radiative centers before being trapped by nonradiative centers. The localized energy states and confinement potentials have been successfully constructed in a wide variety of semiconductors, such as traditional alloy semiconductors,^[^ ^35^, ^36^ ^]^ colloidal quantum dots,^[^ ^37^ ^]^ and 2D atomic crystals^[^ ^38^, ^39^ ^]^ via heteroatom doping, lattice distortion, strain, and moiré superlattice. Inspired by these achievements, here we prepared yellow‐emitting CDs with a near‐unity PLQY of 95%, the highest efficiency for yellow‐emitting CDs till now, which is induced by the localized excitons. The doped pyrrolic nitrogen atoms in the CDs can capture the free excitons and turn them into localized excitons with a large binding energy of 77.9 meV. The short radiative lifetimes of <8 ns and diffusion lengths of <50 nm of the localized excitons are beneficial to a defect‐insensitive emission of CDs through the radiative recombination of the localized excitons. Density functional theory (DFT) calculation verifies that heteroatoms in CDs can effectively perturb the electronic/excitonic structures by adding the confinement potentials and break the forbidden transition of the CDs.^[^ ^40^ ^]^ In addition, the significant structural deformation and highly localized potentials endow the CDs with a large Stokes shift of 0.39 eV and broad emission band that covers green, yellow, and red regions, which are suitable for high‐quality white LEDs. By incorporating CDs into polystyrene (PS), a flexible light‐converted film has been demonstrated with a solid‐state emission efficiency of 84% and excellent physicochemical stability. Employing the CD‐based light‐converted film as luminophore and a blue LED chip as an excitation source, WLEDs have been fabricated. Notably, the WLED shows an LE of 140 lm W^−1^ with a color coordinate of (0.321, 0.338) in CIE 1931 chromaticity coordinates, which outperforms the previous QD‐based WLEDs. Furthermore, a proof‐of‐concept flat‐panel illumination system with a lighting size of more than 100 cm^2^ has been fabricated for the first time by integrating the CDs’ composite film and blue LED bars with a light guide plate, which can act as area lights with tunable lighting color. Hence, such CDs with record‐breaking efficient emission are a promising alternative for traditional rare‐earth‐based phosphors in future solid‐state lighting systems.

## 2 Results and Discussion

### 2.1 Synthesis and Characterization of the CDs

**Figure 1:**
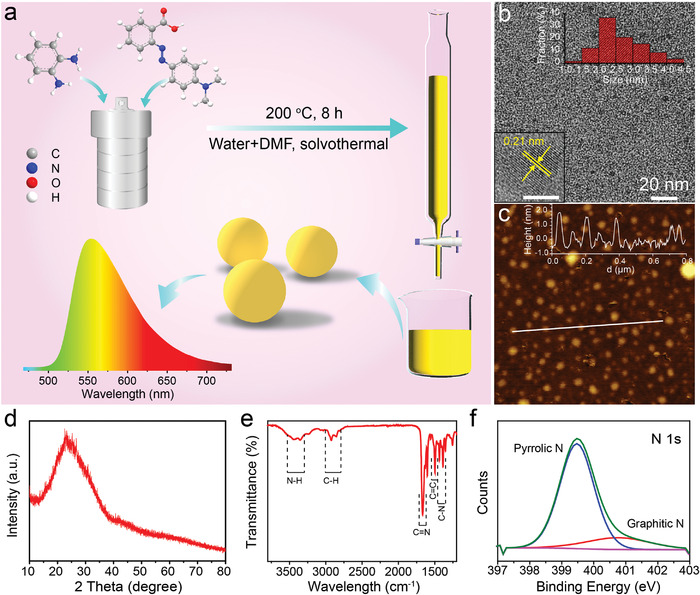
Characterization of the CDs. a) Schematic diagram of the synthesis process of CDs. b) TEM image of the CDs. The insets are HRTEM image and diameter histogram of the CDs. c) AFM image of the CDs. d) XRD pattern of the CDs. e) FT‐IR spectrum of the CDs. f) High‐resolution XPS N 1s spectrum of the CDs.

Methyl red and *o*‐phenylenediamine were selected as a carbon (C) source and nitrogen (N) dopant precursors for their specific molecular structures, which can easily generate sp^2^ backbone and integrate N atoms as sp^3^ species. After an 8 h solvothermal reaction in *N*,*N*‐dimethylformamide (DMF) at 200 °C, CDs with bright yellow emission can be obtained and purified through silica column chromatography. The transmission electron microscopy (TEM) observation indicates that the resulting CDs are well dispersed and have a uniform particle size of about 2.6 nm, as shown in **Figure** **1**b. The high‐resolution TEM (HR‐TEM) image (inset of Figure 1b) shows that the CDs have high crystallinity with the lattice spacing of 0.21 nm, in consistence with the (100) in‐plane lattice spacing of graphitic carbon.^[^ ^41^ ^]^ As evidenced by the atomic force microscopy (AFM) topography shown in Figure 1c, the CDs have a height of around 1.0–2.0 nm. The X‐ray diffraction (XRD) patterns of the CDs exhibit a broad peak at around 23° (Figure 1d), which can be attributed to the (002) planes of graphitic carbon and reflect the existence of layered structure (van der Waals distance ≈ 0.33 nm) in the CDs.^[^ ^2^ ^]^ Fourier transform infrared (FT‐IR) and X‐ray photoelectron energy spectra (XPS) were used to investigate the chemical composition of the CDs (Figure 1e,f). The FT‐IR peaks at 3353 and 1387 cm^−1^ can be attributed to the stretching and bending vibration of sp^3^ N—H and C—N, respectively,^[^ ^9^ ^]^ while the peaks at 1667 and 1532 cm^−1^ can be ascribed to stretching and skeletal vibration of sp^2^ C=N and C=C, respectively.^[^ ^42^ ^]^ Therefore, FT‐IR analysis favors polyaromatic structures with N‐containing groups on their surface. As shown in Figure S1a (Supporting Information), three typical peaks have been observed in the XPS full survey: 284.8 eV (C 1s), 399.2 eV (N 1s), and 531.8 eV (O 1s). In the high‐resolution XPS spectra (Figure S1b, Supporting Information), the C 1s band consists of three peaks at 284.8, 285.3, and 288 eV, corresponding to sp^2^ carbons (C—C/C=C), sp^3^ nitrous carbons (C—O/C—N), and carbonyl carbons (C=O), respectively.^[^ ^42^ ^]^ The N 1s analysis reveals the existence of pyrrolic N (399.5 eV) and graphitic N (400.8 eV),^[^ ^11^ ^]^ as shown in Figure 1f. As pyrrolic N (82.70% of total nitrogen content) dominates the doped N, the CDs may contain substantial sp^3^‐conjugated pyrrole groups at the edge of the graphite structure.

### 2.2 Optical Properties of the CDs

**Figure 2:**
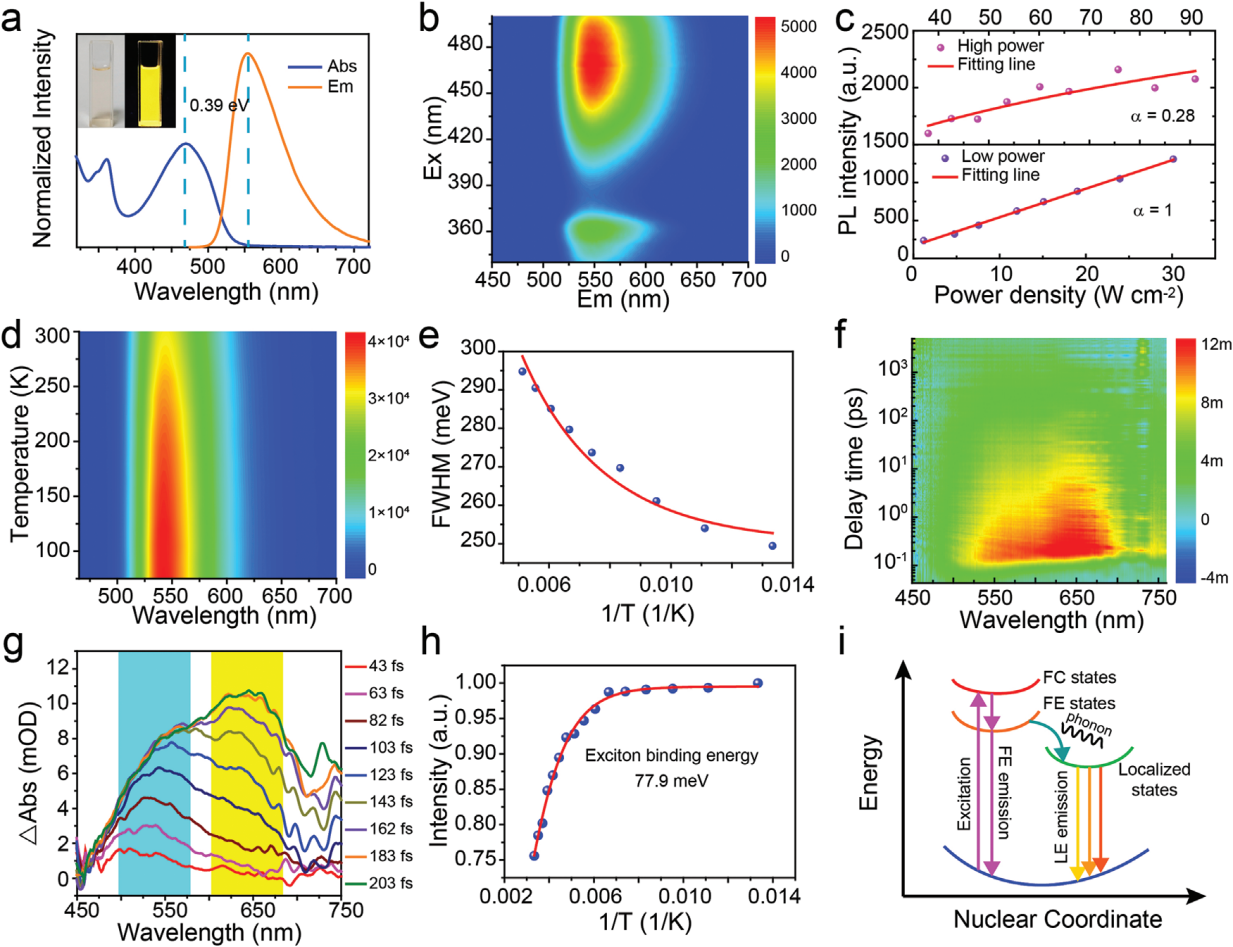
Optical properties of the CDs. a) Absorption and PL spectra of the CDs with optical images in the inset. b) Excitation–emission matrix of the CDs. c) Power‐dependent PL spectra of the CDs. The red solid lines are power‐law fits for the high‐power and low‐power regions. d) Temperature‐dependent PL spectra of the CDs. e) Fitting result of FWHM as a function of temperature. f) TA spectra of the CDs. g) TA spectra with different delay times. h) Fitting results of the integrated intensity as a function of temperature. i) Schematic diagram of the excitonic luminescence in CDs. Here, FC stands for free carrier, FE stands for free exciton, and LE stands for localized exciton.

The CDs distributed in toluene exhibit a broad emission band ranging over green, yellow, and red regions (**Figure** **2**a). A broad absorption band from 400 to 550 nm is observed, corresponding to the n → π* transitions of CDs.^[^ ^11^ ^]^ Besides, the CDs have a typical giant Stokes shift of 0.39 eV, which can effectively reduce reabsorption of the CDs. Intriguingly, the CDs achieve a PLQY of 95% with a deviation of <3.7% among the ten batches of samples (Figures S2–S4 and Table S1, Supporting Information), which is the highest value ever reported for yellow‐emitting CDs. Although solvent thermal treatment of citric acid and urea can manufacture CDs with a PL QY of 92%, the QY of the crude product is only 53%. Cumbersome column chromatography separation means must be used in order to obtain such an efficient yellow CDs, which are very detrimental to their mass production.^[^ ^43^ ^]^ The excitation–emission matrix in Figure 2b reveals a stable emission center at around 552 nm as the excitation wavelength ranges from 330 to 500 nm, i.e., the emission of CDs is independent of the excitation wavelength.^[^ ^44^ ^]^ To further investigate the luminescence mechanism, time‐resolved PL spectra of the CDs were collected, as shown in Figure S5 (Supporting Information). The results show that the emissions ranging from 500 to 600 nm all exhibit single‐exponential decay characteristics with a PL lifetime (*τ*_PL_) varying from 4.5 to 6.0 ns, which implies that the broad emission comes from the same excited state. Above results suggest that all the emissions of the CDs may originate from the radiative recombination of excitonic species.^[^ ^45^ ^]^ Further identification of the excitonic species has been performed by the power‐dependent PL measurements (Figure 2c). The power law of *I*∝*p* ^*α*^ is used to fit the data at the low‐power and high‐power regions, where *I* is the integrated PL intensity, *P* is the excitation power, and *α* ≈ 1 for single exciton complexes and *α* < 1 for localized exciton states.^[^ ^46^ ^]^ At the high‐power regions (30 < *P* < 95 W cm^−2^), the CDs show a sublinear power dependence with an *α* of 0.28. Therefore, the luminescence behavior of the CDs can be ascribed to a typical localized exciton emission. As shown in Figure 2d, the PL of the CDs increases continuously without any shift in the peak wavelength as the temperature decreases from 300 to 75 K, which can be ascribed to be that the exciton dissociation at low temperature, will not change the excitonic bound states. Moreover, PL spectra obtained at 75 K exhibit a full‐width at half‐maximum (FWHM) of 250 meV, slightly less than the value (295 meV) acquired at 300 K, implying that strong coupling between electronic transitions and lattice phonons may exist in the CDs. This may lead to the temperature‐independent fluctuations of transition frequency and homogeneous broadening of emission band. The energy of the optical phonons (ℏ*ω*_phonon_) released from excited state to localized state can be estimated as 29.2 meV through fitting the temperature‐dependent FWHM of the PL peaks^[^ ^40^ ^]^ (Figure 2e). And the time for the free excitons to be localized into bound excitons (*τ* = 2*π*/*ω*_phonon_) can be calculated as 141.7 fs, showing an ultrafast transition process from free excitonic states to localized states.

### 2.3 Origin of High‐Efficient Luminescence in the CDs

To gain more insight into the transition dynamic process of the excitons in the CDs, femtosecond transient absorption (fs‐TA) measurement was carried out with a pump wavelength of 365 nm (Figure 2f). The CDs present a broad photoinduced absorption in the range of 450–750 nm. As the delay time increases from 43 to 203 fs, the absorption band at around 530 nm enhances gradually and the intensity reaches saturation at about 143 fs (Figure 2g). Besides, with a delay time of 103 fs, a new absorption band emerges at around 630 nm and reaches saturation at about 183 fs. The evolution of the absorption band can be attributed to the localization process of the free excitons. According to the ultrafast dynamics characterization, the time for the free excitons turned into localized bound excitons can be estimated to be about 103 fs, which is consistent well with the calculated value by the cryogenic experiments (141.7 fs). These results indicate that photoinduced free excitons in the CDs can be captured by the exciton trapping sites in ultrashort time and converted to localized excitons. When excitons recombine from the localized states, the probability of radiative recombination is enhanced due to the confinement effect, which is favorable for higher PLQY.^[^ ^35^ ^]^

As neutral quasiparticles, localized excitons show different photophysical behaviors in exciton propagation and recombination, depending on the bound strength of the excitonic states. As mentioned above, a certain amount of nitrogen is doped as conjugated pyrrolic N at the edge of multilayer graphene sheet structure in the CDs, which can serve as localized bound state. The exciton binding energy of the CDs is 77.9 meV by fitting the integrated intensities of temperature‐dependent PL spectra^[^ ^3^ ^]^ (Figure 2h). The large exciton binding energy allows the free excitons to be easily captured by the localized state and turned into localized Frenkel‐type excitons.^[^ ^47^ ^]^ Owing to the high delocalization value for the emissive states, the localized excitons are more likely to undergo radiative recombination and emit photons via the nearby localized radiative recombination centers. Moreover, the radiative lifetimes and diffusion lengths of the localized excitons have also been explored to elucidate the origin of the strong defect‐insensitive emission of the CDs for the first time (Figure S6, Supporting Information). Two competitive processes contribute to the overall emission probability of the CDs, i.e., the radiative recombination via localized radiative centers, and the nonradiative recombination in free states. The radiative lifetime (*τ*_R_) and nonradiative lifetime (*τ*_NR_) of the CDs can be derived via the equation: *η*_int_ = 1/(1+ *τ*_R_/*τ*_NR_), where *η*_int_ is internal quantum yield of the CDs^[^ ^35^ ^]^ (Figure S6a and “Methods” section in the Supporting Information). With the emission wavelengths ranging from 510 to 580 nm, *τ*_R_ varies from 4.8 to 7.6 ns. In comparison to the long maximal *τ*_NR_ of 24 ns (Figure S6a, Supporting Information), the short *τ*_R_ values (<8 ns) suggest the low nonradiative trapping levels and high oscillator strength, which are conducive to the enhancement in the PL efficiency of the CDs. In addition, as another key point of the emission process, the diffusion lengths (*L*_c_) of localized excitons in CDs have been investigated by cathodoluminescence (CL) mapping measurement (Figure S6b–d, Supporting Information). The toluene solution of CDs (2 mg mL^‒1^) was dip‐coated onto the silicon substrate to form a uniform CD film. As a control, a bare silicon substrate was also measured, which shows no bright regions (Figure S6b, Supporting Information). The CL emission can be observed from the CD film located in the bright zones, which is the excitonic emission excited by high‐energy electron (Figure S6c, Supporting Information). The diffusion length *L*_c_ determined from the shortest half width of the dark zone between two neighboring bright zones is about 50 nm, which is almost the spatial resolution of the CL system (Figure S6c,d, Supporting Information). The short diffusion length *L*_c_ of the CDs (≈50 nm) evidences the efficient localization of excitons by radiative recombination centers before being captured by nonradiative centers, in favorable for the defect‐insensitive emission of the CDs.^[^ ^35^ ^]^

The most important features for the CDs are ultrahigh PLQY and large Stocks shift. Figure 2i depicts the possible origin of the distinct photophysical behavior mediated by the CD exciton species. When the CDs are excited by incident photons, free excitons generated from the ground state can transport to the nearest emissive traps and transform into localized bound excitons through the strong binding interaction with photons. The large relaxation energy released in this process induces the large Stokes shift of the CDs. In addition, the bound excitons can be highly polarized and localized since the doping of pyrrolic N in the conjugated structure of the CDs and provide short radiative lifetime and diffusion length, which may facilitate the transition of emissive decay from these localized sites and eventually induce a high PLQY.

### 2.4 Theoretical Investigation

**Figure 3:**
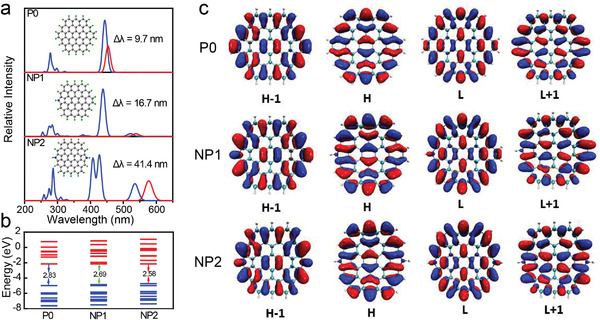
Theoretical calculation of luminescent models in CDs. a) Calculated UV–vis absorption (blue line) and fluorescence (red line) spectra (in the range 200–700 nm) for the nitrogen‐doped CDs (NP1, NP2) and nitrogen‐free system P0 of the same size: carbon (gray), hydrogen (green), nitrogen (blue). b) Relative energy levels of the occupied (blue) and unoccupied (red) molecular orbitals for the nitrogen‐doped and nitrogen‐free CD systems. c) Frontier and near‐frontier molecular orbitals of the nitrogen‐free CD system P0 and nitrogen‐doped CD systems NP1 and NP2. For brevity, the HOMO orbital is denoted as H and the LUMO orbital as L. The red and blue colors in the molecular orbitals represent the positive and negative phases of the molecular orbital wavefunctions.

To further elucidate the luminous mechanism of the yellow‐emissive CDs, the electronic structure and spectral characteristics of the CDs with various pyrrolic nitrogen doping concentration are evaluated using time‐dependent DFT (TD‐DFT) combined with experimental results. The CDs with a round structure of 19 fused benzene rings are used for theoretical analysis, as shown in **Figure** **3**a. Three models are established here: the N‐free (P0) and N‐doped (NP*x* (*x* = 1, 2)) CDs, where *x* corresponds to the number of N atoms in the model. Detailed analysis of the UV–vis absorption and fluorescence properties are conducted for the three models. The calculated long‐wavelength absorption of the N‐free structure P0 features the forbidden S0 → S1 and S0 → S2 transitions at 552.8 and 519.4 nm, respectively, and an intense S0 → S3 transition at 443.5 nm with an oscillator strength (*f*) of 1.506 au (Figure 3a; Table S2, Supporting Information). The transition into the S3 state has major contributions from H → L + 1 (48.8%) and H − 1 → L (48.8%) configurations (Figure 3c; Table S3, Supporting Information). The S3 → S0 fluorescence is assumed for the P0 system, with a calculated wavelength of 453.2 nm. The introduction of pyrrolic N can adjust the symmetry of the frontier orbit, so as to realize the change from the forbidden transition to the allowable transition. When introducing one pyrrolic nitrogen in the CDs, the low‐energy absorption S0 → S1 transition at 542.8 nm is still almost forbidden (*f* = 0.005 au), whereas the S0 → S2 transition at 520.9 nm is allowed with an oscillator strength (*f*) of 0.147 au. The transition into the S2 state has major contributions from H → L (77.3%) and H − 1 → L + 1 (22%). The S2 → S0 fluorescence is assumed for the NP1 system with a wavelength of 537.6 nm. As the number of pyrrolic nitrogen in the model increases to 2, the S0 → S1 transition at 535.3 nm turns allowed with a large oscillator strength (*f*) of 0.403 au, and the transition into S1 state is dominated by H → L (91.5%) contribution. The NP2 system exhibits S1 → S0 fluorescence with a wavelength of 576.7 nm. With the increase of pyrrolic nitrogen, the oscillator strength (*f*) values for the S1 → S0 transitions in these three structures increase gradually from 0 to 0.516, manifesting that the forbidden nature of pure CDs can be broken with the addition of N heteroatoms (Table S4, Supporting Information). Thus, the doping of N breaks the parity‐forbidden S1 → S0 transition by manipulating the symmetry of the wavefunction, which facilitates the formation of localized excitons and enhances the PLQY of CDs greatly.

Above calculation shows that the Stokes shift of the CDs increases from 9.7 to 41.4 nm with the doping of N atoms. A similar trend has been observed in the experimental results of CDs with increasing the pyrrolic N content from 0% to 12.7% (Figure S7, Supporting Information). The H (L) and H − 1 (L + 1) orbits are degenerate in energy to the P0 system. The doping of N breaks the H (L) and H − 1 (L + 1) degeneration and reduces the H–L gap, which in turn allows the low‐energy S0 → S2 and S0 → S1 transitions (Figure 3b). With the increase of pyrrolic N amount, the S0 → S1 transition becomes stronger and a larger Stokes shift is expected owing to a more prominent geometry deformation at the defect sites, in well accordance with the experimental results (Figure S7, Supporting Information). Therefore, the large Stokes shift energies of the CDs can be attributed to the introduction of heteroatoms (pyrrolic N) in the sp^2^‐conjugated structure, by breaking the degeneration of the frontier molecular orbitals, reducing the highest occupied molecular orbital (HOMO)–lowest unoccupied molecular orbital (LUMO) gap of the CDs, and finally resulting in the significantly strengthened low‐energy S0 → S1 transition.

### 2.5 Yellow‐Emitting CDs@PS Composite Films

**Figure 4:**
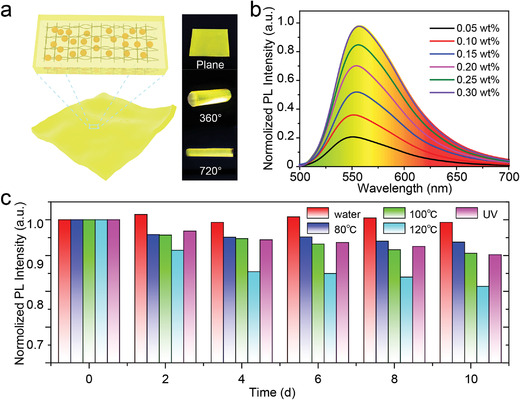
Optical properties of CDs@PS composite film. a) Schematic diagram and photographs of flexible CDs@PS composite film. b) PL spectra of the composite films with different ratios of CD and PS ranging from 0.05 to 0.30 wt%. c) Environmental stability measurement of the composite film in different conditions.

Generally, aggregation‐induced luminescence quenching is a common problem for CDs, which usually leads to quite low emission efficiency in solid state and greatly limits their practical applications.^[^ ^48^, ^49^, ^50^, ^51^ ^]^ To address this issue, an effective way is dispersing CDs into polymer matrices. As a widely used plastic, PS has advantages of low cost, easy processing, and high transparency. Here, CDs@PS composite films are prepared with a facile method to avoid the fluorescence quenching of CDs in solid state. After dispersing the CDs into the matrix of PS, the obtained solid CDs@PS composite film maintains a high PLQY of 84% and a large Stocks shift of 73 nm at 0.05 wt% (Figure S8, Supporting Information). When illuminated by a UV light, the composite film emits bright yellow light. Notably, the composite film has good flexibility and can even be rolled up, which allows it compatible to roll‐to‐roll technology (**Figure** **4**a). When the CD ratio in the composite films increases from 0.05 to 0.30 wt%, the PL intensity increases linearly and the PLQY slightly declines from 83.9% to 80.3% (Figure 4b; Figure S9, Supporting Information). Additionally, the CDs@PS composite film shows good photo‐, thermal‐, and solvent stability. After being irradiated by UV light (0.15 mW cm^‒2^) for 10 days, the PL intensity of the composite film only decreases by about 10% (Figure 4c). After a 10 day heat aging test in ambient atmosphere, the samples heated at 80 and 100 °C retain 93% and 91% of their initial PL intensity, respectively. Even when the heated temperature is raised to 120 °C, the decrease in the PL intensity is still less than 20%. In addition, this composite film exhibits excellent water resistance. After immersed in water for 10 days, no degradation in the PL intensity has been observed. Excellent water resistance enables this film applicable in severe humid environments (Figure S10, Supporting Information).

### 2.6 High‐Efficiency WLED Based on the CDs

**Figure 5:**
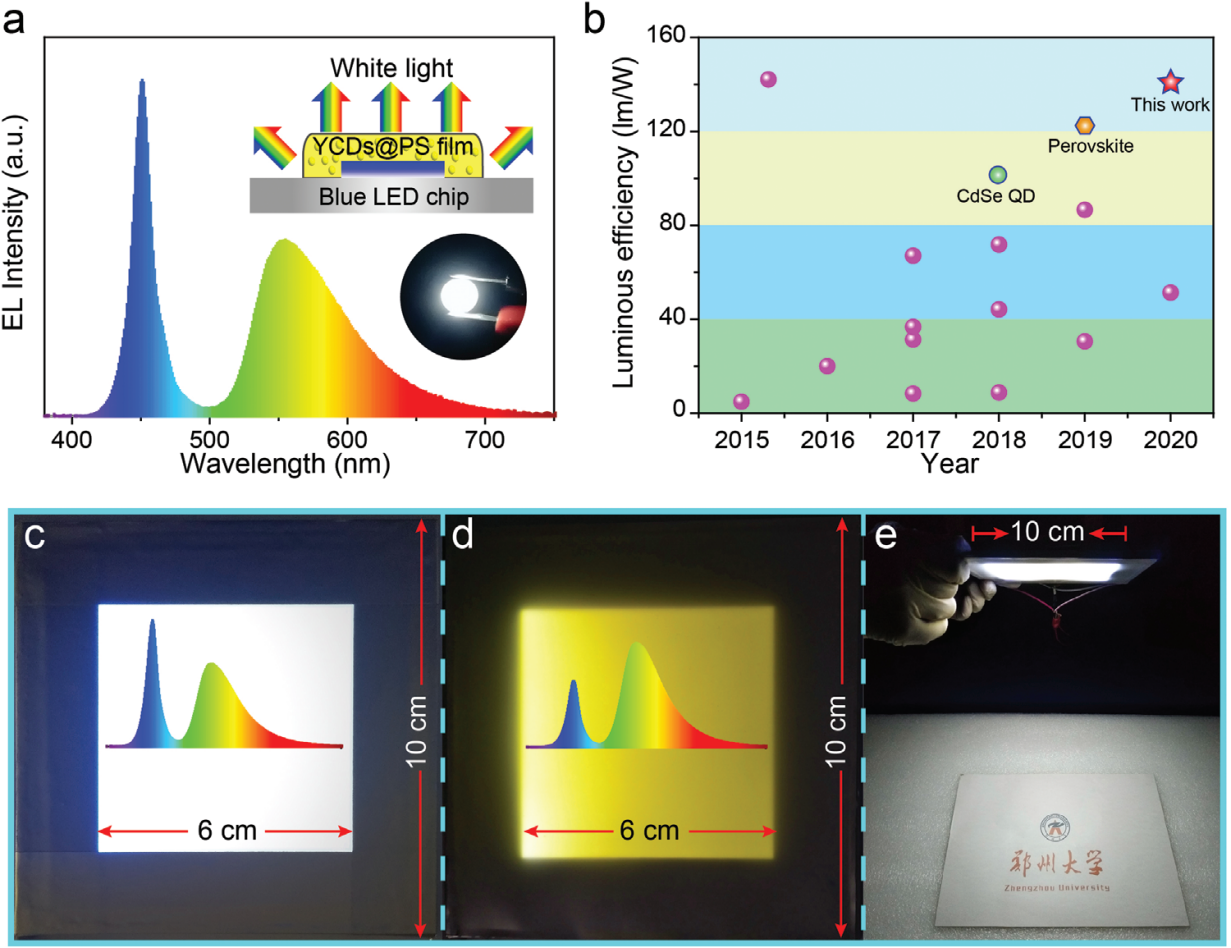
CD‐based WLEDs and flat‐panel illumination system. a) EL spectrum of the CD‐based WLED with a schematic diagram and photo of the WLED in the inset. b) Comparison of the LE of WLEDs based on CDs (purple points) and other materials. A detailed data point is presented in Table S5 (Supporting Information). c,d) Photograph of CD‐based area lights with c) cold and d) warm‐white light. The insets of panels (c) and (d) are the corresponding EL spectra. e) Picture under the flat‐panel illumination system with a light emitting area of 10 cm × 10 cm.

To realize high‐efficiency WLEDs, another key point is to reduce the self‐absorption of the phosphors. As shown in Figure S11a (Supporting Information), the numerical simulation results clearly show the dependence of luminous efficiency (LE) on the two factors, i.e., Stokes shift and PLQY. The high LE of WLEDs is expected from phosphors with both high PLQYs and large Stokes shift, which can effectively reduce emission losses by suppressing self‐absorption. More importantly, when the PLQY is close to unity, well‐engineered Stokes shift becomes increasingly important to realize the good chromatic stability of WLEDs (Figure S11b, Supporting Information). Therefore, with the merits of high PLQY, large Stokes shift, broad emission band, outstanding stability, and good biocompatibility, the CDs can be employed as environmentally friendly phosphors to meet the requirements of efficient WLEDs (Figure S12, Supporting Information). To confirm the above speculation, WLEDs were fabricated by simply coating the CDs@PS composite film on a blue‐emitting GaN chip (Figure S13, Supporting Information). The electroluminescence (EL) spectrum of the WLED shows two emission bands at 450 and 552 nm, corresponding to the blue LED chip and the emission of the CDs (**Figure** **5**a; Figure S14, Supporting Information). As shown in the inset of Figure 5a, the WLED can emit bright white light with coordinates of (0.321, 0.338), close to that of the standard white light (Figure S15, Supporting Information). Notably, an ultra‐high LE of 134 lm W^−1^ with fluctuations lower than 6.0% can be achieved with a drive current of 5 mA, which is much higher than the CD‐based LEDs ever reported^[^ ^21^, ^24^, ^30^, ^31^, ^52^, ^53^, ^54^, ^55^, ^56^, ^57^, ^58^, ^59^ ^]^ (Figure 5b; Figure S16, Supporting Information). The CD‐based WLEDs show a nearly linear increase of EL intensity and almost unchanged emission peak wavelength when the drive current increases to 50 mA (Figure S17a, Supporting Information). An LE of over 100 lm W^−1^ can be kept in the CD‐based WLED even under a high current of 50 mA, guaranteeing good performance at high light output conditions (Figure S17b, Supporting Information). Figure 5b summarizes the LE performance of WLEDs with various phosphors. To the best of our knowledge, WLEDs utilizing the perovskite assisted with commercial rare‐earth phosphors and CdSe quantum dots present optimal LE performances of 124 and 105 lm W^−1^, respectively.^[^ ^23^, ^28^ ^]^ In our study, the CD phosphor further improves the LE of the WLEDs to ≈140 lm W^−1^. The CD‐based WLEDs show a good device stability as well, whose luminance undergoes just a 27.0% degradation after continuous operation of 12 h (Figure S18, Supporting Information). Furthermore, the WLEDs with different correlated color temperature (CCT) from 4000 to 9373 K can be fabricated by gradually adjusting the concentration of the CDs from 0.10 to 3.0 wt% (Figure S19, Supporting Information). These white‐light devices cover from cold white to warm white region, which could be applied in various illumination occasions. The color rendering index (CRI) of the WLED in our work is around 60. This low CRI value is mainly ascribed to the single‐phosphor structure of the WLED because the emission spectrum of the yellow‐emissive CDs cannot cover the whole visibility region.

### 2.7 Large‐Scale White Area Light

As a proof‐of‐concept prototype, a large‐scale white area light source based on CDs has also been fabricated to explore the potential of the CDs in the flat‐panel illumination system. The detailed structure is shown in Figure S20 (Supporting Information). A light guide plate with the CDs@PS composite film on its top is the major downconversion luminescence part; a light reflection panel and a light diffuser are attached on the bottom and top of the system, respectively. Meanwhile, one or two blue LED bars are installed on the side as an excitation light source. The light guide plate guarantees the blue light from LED chips uniformly emit out of the surface. Then the composite film will be excited and give out bright yellow light. Finally, taking the advantage of the light diffuser plate, the blue light and yellow emission can be well mixed to send out white light from the top surface (Figure 5c). A 6 cm × 6 cm flat‐panel illumination system has been prepared with color coordinates at (0.302, 0.356) and a CCT of 6814 K in the cold light region. Considering the harmful effect of excessive blue light to human eyes, a warm‐white area light source with color coordinates of (0.362, 0.447) and a CCT of 4797 K can be conveniently fabricated by increasing the concentration of the CDs (Figure 5d). In addition, the CD‐based area light source illustrates good stability. When kept operating at a driven current of 20 mA for 24 h, there is only a 13% decrease in the emission intensity, which can satisfy the daily requirement for illumination (Figure S21, Supporting Information). To verify the practical application of the area light, the device was placed in the air, and it can still work after 1 year (27.8% decrease of the emission intensity) (Figure S21, Supporting Information). To further demonstrate its application as indoor lighting, we have fabricated the CD‐based flat‐panel illumination system with a light emitting area of 10 cm × 10 cm (Figure 5e). Under the illumination of the flat panel, the document below it can be brightly lit up with perfect color rendition.

### 2.8 Electroluminescent Device

By virtue of the localized exciton emission, it is promising to fabricate CD‐based electroluminescent devices with low turn‐on voltage. As a proof of concept, electroluminescent devices based on the CDs have been fabricated with an inverted structure. Detailed structure and performance of the electroluminescent device are shown in Figure S22 (Supporting Information). As expected, the electroluminescent device has a low turn‐on voltage of about 3.0 V, which indicates the low charge injection barrier in the device. A possible mechanism is that under the applied electric field, the charges can transport and directly inject to the highly localized states of the CD‐based luminescent layer to form the emissive located excitons, which will decrease in the charge injection barrier and further lower the turn‐on voltage. Therefore, the located exciton‐mediated emission may also provide a universal strategy to fabricate a low‐power‐consumption electroluminescent device with low turn‐on voltage.

## 3 Conclusion

We here developed a heteroatom‐doping solvothermal method to synthesize yellow‐emitting CDs with a PLQY up to 95%, which is the highest value among the ever reported yellow‐emitting CDs. Both experimental and TD‐DFT calculation results clarify two main reasons for the high PLQY of CDs: enhanced radiative recombination of highly localized excitons because of the confinement effect; the breakdown of forbidden transition by introducing N atoms. The short radiative lifetimes (<8 ns) and diffusion lengths (<50 nm) of the CDs are beneficial to the localization of excitons at the radiative recombination centers and contributing to a high‐efficiency defect‐insensitive emission. By embedding the CDs into PS, flexible yellow‐emitting CDs@PS composite films have been fabricated. WLEDs with color coordinates of (0.321, 0.338) and a maximum LE of 140 lm W^−1^ have been prepared by coating the composite film on a blue chip, which is the most efficient WLED ever reported among the colloidal nanocrystal‐based WLEDs. Moreover, based on the composite film, we have demonstrated a large‐scale area light source with an effective light emitting area of 100 cm^−2^. In addition, electroluminescent device based on the CDs presents a low turn‐on voltage of about 3.0 V owing to the low charge injection barrier induced by the highly localized states of the CDs as the emitter layer. The results reported in this paper provide a universal strategy to design and synthesis efficient CDs, and promise their applications in optoelectronic devices.^[^ ^60^ ^]^

Lastly, the mechanism of localized excitons on regulating photo‐ and electroluminescent performances of CDs is quite complicated and needs further study. In spite of this, the changes in luminescence property induced by heteroatom doping are clearly evident from the above experiments, offering a new design option for high‐performance CD‐based luminescent devices.

## 4 Experimental Section

The “Experimental Section” is available in the Supporting Information.

## Conflict of Interest

The authors declare no conflict of interest.

## Author Contributions

Q.L., Q.N., and C.N. contributed equally to this work. Q.L., L.D., and C.‐X.S. designed the project. J.W., Q.N., and C.N. performed the experiments and calculation. All authors analyzed and interpreted the data, contributed to the writing of the manuscript, discussed the results and implications, and edited the manuscript at all stages.

## Supporting information

Supporting Information

## Data Availability

The data that support the findings of this study are available from the corresponding author upon reasonable request.
