# Supplementary material for: Carbon Nanodots with Nearly Unity Fluorescent Efficiency Realized via Localized Excitons
Source: Adv Sci (Weinh). 2022 Aug 24;9(30):2203622. doi: 10.1002/advs.202203622 (PMC9596859; doi:10.1002/advs.202203622)
Supplement: Supplementary file 1 — Supporting Information [file ADVS-9-2203622-s001.pdf]

Supporting Information

Carbon Nanodots with Nearly Unity Fluorescent Efficiency Realized via Localized Excitons

Qing Lou, Qingchao Ni, Chunyao Niu, Jianyong Wei ,Zhuangfei Zhang, Weixia Shen, Chenglong Shen, Chaochao Qin, Guangsong Zheng, Kaikai Liu, Jinhao Zang, Lin Dong* and Chong-Xin Shan**

These authors contributed equally: Qing Lou, Qingchao Ni, Chunyao Niu

Experimental Section

4.1. Materials

Methyl red (99%, Aladdin), o-phenylenediamine (98%, Aladdin), and polystyrene (99%, Aladdin) were used as precursors for the preparation of CDs and composite films. Blue GaN LED chips (450 nm, Shenzhen Looking Long Technology Co., Ltd) were used as the excitation source for the WLED.

4.2. Synthesis of Yellow-Emitting CDs

Methyl red (0.1 g) and o-phenylenediamine (0.1 g) were dissolved in water–*N*,*N*-Dimethylformamide (DMF) mixture (10 ml, vol/vol = 1:1). Then the mixture was transferred into a Teflon-lined stainless-steel autoclave (25 ml) and heated at 200 °C for 8 hours. After the reaction, the reactor was cooled to room temperature. The solution was purified via silica column chromatography using dichloromethane as the eluent. Then, the obtained solution was dried in a vacuum oven at 60 °C. Finally, the yellow-emitting CDs solution was obtained by adding toluene as solvent.

4.3. Synthesis of CD1, CD2 and CD3

For CD1, 0.1 g of phloroglucinol was dissolved in 10 ml of alcohol. Stir thoroughly to

dissolve the phloroglucinol completely. Then add the reactant to a 25-ml Teflon reactor and heat it at 200 °C for 5 hours. After the reaction, the reactant was cooled to room temperature, then CD1 was obtained. The synthesis method of CD2 and CD3 is similar to that of CD1. The raw materials of CD2 are 0.1-g phloroglucinol and 0.1-g o-phenylenediamine, and the raw material for CD3 is 0.1-g o-phenylenediamine.

4.4. Fabrication of the Composite Film with CDs In PS

To fabricate CDs@PS composite films with different mass ratio from 0.05 wt. % to 0.30 wt. %, 0.5 g of PS was added into 5-30 ml of CDs solution (0.05 mg ml^{-1} in toluene). The mixture was heated and continuously stirred at 60 °C until the PS was dissolved. Then, the homogeneous mixture was poured onto a piece of glass. After the evaporation of the solvent at 60 °C, CDs@PS composite films were obtained on the glass.

4.5. Fabrication of LED Devices

CDs and PS (0.10 wt. %) mixture in toluene solution was dropped on the LED chip carefully, and heated the LED chip at 45 °C to control the evaporation rate of solvent at a low level. After the evaporation of the solvent, the WLED was obtained.

4.6. Fabrication of Flat Panel Illumination System

Firstly, a light reflecting film was attached to the back surface of a light guide plate, and a light bar that consists of several blue GaN LED chips was installed on one side of the light guide plate (Epistar Corporation, China). Then, full sides of the light guide plate were covered with reflective tape. To obtain a uniform CDs@PS composite film on the top surface of the light guide plate, a blade coating was used. Certain amount of the mixture of CDs and PS was dropped on one side of the top surface of the light guide plate and then the blade was pushed forward with a low and constant speed to form a layer of wet film on the surface. After heated about 2 h at 45 °C, the solvent in the composite film could be completely moved and a uniform film generated on the top surface of the plate. Finally, a light diffuser plate was attached on the composite film and the CD-based flat panel illumination system was obtained.

4.7. Femtosecond Transient Absorption

Helios pump-probe system (Ultrafast Systems LLC) coupled with an amplified femtosecond laser system (Coherent, 35 fs, 1 kHz, 800 nm) was used. The probe pulses (from 450 to 760 nm) were generated by focusing a small portion (around 10 μJ) of the fundamental 800-nm laser pulses into a 1-mm CaF_2. The 365-nm pump pulses were generated from an optical parametric amplifier (TOPAS-800-fs).

4.8. Preparation of CD-Based Electroluminescence Device

Transparent indium tin oxide (ITO) glass substrates were ultrasonically cleaned and plasma treated for 10 min in the different organic solvents (acetone, ethanol and deionized water) as a cathode. And then, ZnO nanoparticles (30 mg ml^{-1}) as an electron transport layer were spin-coated onto ITO substrate at a speed of 2000 rpm with a heat treatment at 120 °C for 30 min. were deposited in the glove box. After cooling, the CDs dispersed in toluene solutions (8 mg ml^{-1}) as the luminous layer were spin-coated on the ZnO layer at the speed of 2000 rpm, then annealed at 80 °C for 30 min in glovebox. And finally, 4,4′-bis(carbazol-9-yl)biphenyl (CPB) hole transport layer and MoO_3/Al double-layered anode were deposited by step-by-step thermal evaporation. With a help of metal mask, the effective emission area of the device is kept at about 4 mm^2.

4.9. Characterization

FEI Talos F200 transmission electron microscopy was used to obtain high-resolution TEM images of the CDs. The X-ray diffraction (XRD) patterns of the CDs were recorded by using Bruker D8 Discover (Germany) X-ray diffractometer. The X-ray photoelectron energy spectra (XPS) were collected using a Kratox AXIS HIS 165 spectrometer with a monochromatized Al KR X-ray source. Fourier transform infrared (FT-IR) spectra were measured by a Thermo Nicolet iz 10 spectrometer. To collect the PL spectra of the CDs solution, a Hitachi F-7000 spectrometer was used. The UV-vis absorption and transmittance spectra were recorded by Hitachi UH-4150 spectrometer. Fluorescence lifetimes and low-temperature fluorescence were measured using Horiba FL-322 using a 405-nm NanoLED as the excitation source. The power-dependent photoluminescence was obtained on a Mr350 Raman

spectrometer (SoL Instrument) with the excitation source of 325-nm He-Cd laser (Kimmon, Japan). The absolute fluorescence quantum yield measurement of the CDs was conducted using an Andor Shamrock 500i-A spectrometer (Oxford Instruments, UK) equipped with a 30-cm integrating sphere and a 405 nm LED light source. The photoelectric properties of the CDs based LED were measured by a sw2000s integrating sphere spectroradiometer with a 10 W reference lamp for calibration (Mulansphere Co. Ltd, China). Room-temperature panchromatic cathodoluminescence (JOLT, Delmic Company) mapping monitoring the spectral range from 320 to 900 nm was taken through a JEOL scanning electron microscope (SEM) with the electron beam acceleration energy of 10 keV.

4.10. Calculation of optical phonon energy ($\hbar\omega_{phonon}$)

$\hbar\omega_{phonon}$ can be obtained by fitting the temperature-dependent full-width at half-maxima (FWHM) of PL peaks using the following relation (1):

$$\mathrm{FWHM} = 2.36\sqrt{S}\hbar\omega_{phonon}\sqrt{\coth(\frac{\hbar\omega_{phonon}}{2k_BT})} \quad (1)$$

where S is Huang-Rhys factor and k_B is Boltzmann constant [1].

4.11. Lifetime of radiative and nonradiative recombination

The photoluminescence decay spectra of the CDs with monitored emissions ranging from 500 to 600 nm all can be fitted by a single-exponential function with a variable PL lifetime (τ_{PL}). The radiative lifetimes (τ_R) and effective non-radiative lifetimes (τ_{NR}) for the localized exciton emission in CDs are derived from the experimental results of τ_{PL} and internal quantum yield (η_{int}) using the following equations (2 and 3):

$$\frac{1}{\tau_{PL}} = \frac{1}{\tau_R} + \frac{1}{\tau_{NR}}$$

(2)

$$\eta_{int} = \frac{1}{1+{}^{\tau_R}/_{\tau_{NR}}}$$

(3)

Where η_{int} for the CDs is approximated as I_{PL} (300 K)/ I_{PL} (75 K) [2].

4.12. Theoretical calculations

Full geometry optimizations of all considered models were performed using density functional theory ((DFT) with the Becket three-parameter hybrid density functional ((B3LYP) [3] with the D3 empirical dispersion correction by Grimme *et al.* [4] and the 6-311G(d) basis set [5]. All the optical properties of all considered models were calculated using time-dependent density functional theory method (TDDFT) with B3LYP function and 6-311G(d) basis set. The excited state was optimized in vacuum to calculate the emission energy (wavelength) which is the energy difference between the ground and the excited state. The Gaussian09 software package has been used throughout this work [6].

Cartesian coordinates of the calculation models:

P0:

H	2.48218600	6.75253100	0.00000000
H	4.60640100	5.52631700	0.00000000
C	3.66395300	4.98444600	0.00000000
H	5.84326500	3.37384200	0.00000000
C	4.90140700	2.82993700	0.00000000
H	7.08887000	1.22622700	0.00000000
C	6.14845900	0.68095500	0.00000000
H	7.08889500	-1.22624400	0.00000000
H	0.00000200	6.74754400	0.00000000
C	2.48447700	5.66543600	0.00000000
C	1.22337700	4.98145400	0.00000000
C	3.70222900	3.55032600	0.00000000
C	2.46132100	2.84006100	0.00000000
C	4.92561700	1.43127900	0.00000000
C	3.69014000	0.71159000	0.00000000
C	6.14845900	-0.68095800	0.00000000
C	4.92561700	-1.43128000	0.00000000
H	5.84328600	-3.37385500	0.00000000

H	-2.48218200	6.75253300	0.00000000
C	0.00000000	5.65990100	0.00000000
C	-1.22337500	4.98145500	0.00000000
C	1.22880400	3.55170400	0.00000000
C	0.00000000	2.84396800	0.00000000
C	2.46288600	1.42204200	0.00000000
C	1.22722100	0.70853200	0.00000000
C	3.69014000	-0.71159100	0.00000000
C	2.46288600	-1.42204300	0.00000000
C	4.90140600	-2.82994000	0.00000000
C	3.70223000	-3.55032700	0.00000000
H	4.60642400	-5.52633000	0.00000000
C	-2.48447600	5.66543800	0.00000000
C	-3.66395300	4.98444700	0.00000000
C	-1.22880300	3.55170500	0.00000000
C	-2.46132100	2.84006100	0.00000000
C	0.00000000	1.41714700	0.00000000
C	-1.22722000	0.70853200	0.00000000
C	1.22722000	-0.70853200	0.00000000
C	0.00000000	-1.41714700	0.00000000
C	2.46132100	-2.84006100	0.00000000
C	1.22880400	-3.55170500	0.00000000
C	3.66395400	-4.98444800	0.00000000
C	2.48447600	-5.66543800	0.00000000
H	-4.60642100	5.52632700	0.00000000
C	-3.70223000	3.55032600	0.00000000
C	-4.90140600	2.82993900	0.00000000
C	-2.46288600	1.42204300	0.00000000
C	-3.69014000	0.71159100	0.00000000
C	-1.22722100	-0.70853200	0.00000000
C	-2.46288600	-1.42204200	0.00000000
C	0.00000000	-2.84396800	0.00000000
C	-1.22880400	-3.55170500	0.00000000
C	1.22337500	-4.98145500	0.00000000
C	0.00000000	-5.65990100	0.00000000
H	2.48218200	-6.75249700	0.00000000
H	-5.84327700	3.37385000	0.00000000
C	-4.92561700	1.43128000	0.00000000
C	-6.14845900	0.68095700	0.00000000
C	-3.69014000	-0.71159000	0.00000000
C	-4.92561800	-1.43127900	0.00000000
C	-2.46132100	-2.84006100	0.00000000
C	-3.70223000	-3.55032700	0.00000000
C	-1.22337700	-4.98145400	0.00000000
C	-2.48447700	-5.66543700	0.00000000
H	-0.00000200	-6.74750800	0.00000000
H	-7.08888200	1.22623600	0.00000000
C	-6.14845900	-0.68095600	0.00000000
H	-7.08888300	-1.22623500	0.00000000
C	-4.90140700	-2.82993800	0.00000000
H	-5.84327400	-3.37384600	0.00000000
C	-3.66395400	-4.98444700	0.00000000
H	-4.60640400	-5.52632000	0.00000000

H	-2.48218600	-6.75249400	0.00000000

NP1:

H	-2.32795600	-6.76497700	0.00000000
H	-4.44706400	-5.56035000	0.00000000
C	-3.51410700	-5.00570600	0.00000000
H	-5.72117200	-3.39448900	0.00000000
C	-4.78350500	-2.84675700	0.00000000
H	-6.98341500	-1.22049400	0.00000000
C	-6.04121700	-0.68176200	0.00000000
H	-6.98342900	1.22102800	0.00000000
H	0.18654900	-6.74474300	0.00000000
C	-2.32886000	-5.67955300	0.00000000
C	-1.06180000	-4.99458100	0.00000000
C	-3.57815100	-3.56629200	0.00000000
C	-2.34359300	-2.85292600	0.00000000
C	-4.82014900	-1.44143000	0.00000000
C	-3.59335200	-0.71529000	0.00000000
C	-6.04121000	0.68232700	0.00000000
C	-4.82017100	1.44197500	0.00000000
H	-5.72111500	3.39510300	0.00000000
H	2.77701700	-6.64456500	0.00000000
C	0.17730600	-5.65872800	0.00000000
C	1.40270400	-4.95953300	0.00000000
C	-1.09988100	-3.56373100	0.00000000
C	0.11684700	-2.84847700	0.00000000
C	-2.36903700	-1.43120700	0.00000000
C	-1.14771900	-0.71045900	0.00000000
C	-3.59334500	0.71579100	0.00000000
C	-2.36906600	1.43168500	0.00000000
C	-4.78347100	2.84730700	0.00000000
C	-3.57816200	3.56684000	0.00000000
H	-4.44706000	5.56096300	0.00000000
C	2.71412200	-5.56077500	0.00000000
C	3.88061300	-4.83178200	0.00000000
C	1.36551200	-3.54123000	0.00000000
C	2.58473500	-2.80853700	0.00000000
C	0.07041400	-1.42266000	0.00000000
C	1.27168700	-0.70494400	0.00000000
C	-1.14770200	0.71085300	0.00000000
C	0.07043700	1.42301600	0.00000000
C	-2.34359300	2.85342300	0.00000000
C	-1.09991600	3.56419600	0.00000000

C	-3.51412400	5.00627100	0.00000000
C	-2.32883700	5.68007100	0.00000000
H	4.82741300	-5.36329900	0.00000000
C	3.89224600	-3.39155700	0.00000000
C	5.08968400	-2.57677600	0.00000000
C	2.49217500	-1.41625500	0.00000000
C	3.65641600	-0.68816700	0.00000000
C	1.27169800	0.70511000	0.00000000
C	2.49219200	1.41667800	0.00000000
C	0.11663600	2.84884500	0.00000000
C	1.36526400	3.54146800	0.00000000
C	-1.06178800	4.99513600	0.00000000
C	0.17738400	5.65918100	0.00000000
H	-2.32792300	6.76550100	0.00000000
H	6.05132600	-3.07763000	0.00000000
C	4.96757800	-1.17589700	0.00000000
C	3.65636200	0.68924900	0.00000000
C	4.96979800	1.17268300	0.00000000
C	2.58386000	2.80885400	0.00000000
C	3.89197600	3.38996000	0.00000000
C	1.40263900	4.95986500	0.00000000
C	2.71449000	5.56010200	0.00000000
H	0.18676900	6.74520900	0.00000000
H	6.78875400	-0.00090400	0.00000000
C	5.08811900	2.57585300	0.00000000
H	6.04964200	3.07711800	0.00000000
C	3.88061200	4.83050000	0.00000000
H	4.82752800	5.36191000	0.00000000
H	2.77842300	6.64384300	0.00000000
N	5.78303000	-0.00179500	0.00000000

NP2:

H	7.06809600	1.14011900	0.00000000
H	7.02895000	-1.26551400	0.00000000
C	6.08066200	-0.73613600	0.00000000
H	5.69143600	-3.52014200	0.00000000
C	4.76548100	-2.95538900	0.00000000
H	5.80056700	3.43819700	0.00000000
C	6.10294800	0.64251100	0.00000000
C	4.91232200	1.46695500	0.00000000
C	4.85538600	-1.50362400	0.00000000
C	3.68566700	-0.68417600	0.00000000
C	3.49951900	-3.58706600	0.00000000

C	2.41109900	-2.71266600	0.00000000
C	1.46614300	-4.79207600	0.00000000
H	0.35498600	-6.68268200	0.00000000
H	4.39320200	5.65277800	0.00000000
C	4.86606100	2.88427800	0.00000000
C	3.64515400	3.60458800	0.00000000
C	3.68670700	0.74688100	0.00000000
C	2.45446900	1.45436400	0.00000000
C	2.45086000	-1.33960000	0.00000000
C	1.21709200	-0.66680100	0.00000000
C	1.22173800	-3.41746500	0.00000000
C	-0.00175900	-2.79302600	0.00000000
C	0.30418600	-5.59926700	0.00000000
C	-1.01241300	-4.98101100	0.00000000
H	-2.26496300	-6.77320600	0.00000000
C	3.49325400	5.04513200	0.00000000
C	2.27349900	5.68743400	0.00000000
C	2.42497100	2.87493100	0.00000000
C	1.16917800	3.56092300	0.00000000
C	1.22833700	0.72753600	0.00000000
C	0.00000000	1.38692000	0.00000000
C	0.00044900	-1.38773300	0.00000000
C	-1.22804700	-0.72807400	0.00000000
C	-1.16957500	-3.56148400	0.00000000
C	-2.42533600	-2.87526900	0.00000000
C	-2.27396400	-5.68721000	0.00000000
C	-3.49398400	-5.04477700	0.00000000
H	2.26426400	6.77340100	0.00000000
C	1.01140800	4.98123800	0.00000000
C	-0.30384300	5.59999000	0.00000000
C	0.00188200	2.79237900	0.00000000
C	-1.22151900	3.41718000	0.00000000
C	-1.21668400	0.66601400	0.00000000
C	-2.45028900	1.33921200	0.00000000
C	-2.45427500	-1.45465500	0.00000000
C	-3.68617500	-0.74674400	0.00000000
C	-3.64547400	-3.60450900	0.00000000
C	-4.86655500	-2.88332600	0.00000000
H	-4.39388200	-5.65247400	0.00000000
H	-0.35571900	6.68334300	0.00000000
C	-1.46630900	4.79214200	0.00000000
C	-2.41090200	2.71275000	0.00000000
C	-3.49951500	3.58581700	0.00000000
C	-3.68468300	0.68421500	0.00000000

C	-4.85480200	1.50437400	0.00000000
C	-4.91215800	-1.46656500	0.00000000
C	-6.10287600	-0.64162900	0.00000000
H	-5.80122300	-3.43695500	0.00000000
H	-3.41232900	5.75364800	0.00000000
C	-4.76585100	2.95501400	0.00000000
H	-5.69106700	3.52085300	0.00000000
C	-6.08053500	0.73672600	0.00000000
H	-7.02851500	1.26655600	0.00000000
H	-7.06798500	-1.13933200	0.00000000
H	3.41166300	-5.75654500	0.00000000
N	-2.89854800	4.89047500	0.00000000
N	2.89809400	-4.89010500	0.00000000

4.13. Simulation of white LED

The calculation is based on the reference [7]. For a down-conversion white LED, the spectral overlap between the absorption of down-conversion materials and the electroluminescence of the LED chip results in photoluminescence of the down-conversion materials. Then the total output power spectral density is generated by combination of photoluminescence of the down-conversion materials and transmitted incoming photon flux. Therefore, the output power spectral density can be calculated by following equations (4-6):

$$S_{out}(\lambda) = S_{in}(\lambda)exp[-\alpha(\lambda)D] + WC(\lambda)P(\lambda) \quad (4)$$

$$W = Q\int S_{in}(\lambda)\{1 - exp[-\alpha(\lambda)D]\}d\lambda \quad (5)$$

$$C(\lambda) = \frac{exp[-\alpha(\lambda)D]}{1 - Q\int P(\lambda)\{1 - exp[-\alpha(\lambda)D]\}d\lambda}$$

(6)

In the equations, $S_{out}(\lambda)$ is the output power spectral density and $S_{in}(\lambda)$ is the input power spectral density. $P(\lambda)$ is the normalized PL spectrum and Q is the photoluminescent quantum yield. $\alpha(\lambda)$ is the absorption coefficient and D is optical path length. W is corresponded to the emission strength, when only emission

(radiative recombination) is considered. C(λ) is the spectral multiplication factor to the overall emission due to reabsorption and inter-absorption, which is the simplified form of the sum of infinitely many reabsorption and inter-absorption cycles.

From the equations above, output power spectral density ($S_{out}(\lambda)$) of the WLED can be obtained. Then, the luminous efficiency (LE) of the WLED can be calculated by the following equation (7):

$$LE = \frac{683 \cdot \int_0^\infty S_{out}(\lambda) \cdot V(\lambda) d\lambda}{P_{in}} \tag{7}$$

Where the $V(\lambda)$ is luminosity function and P_{in} is the input power.

Figures

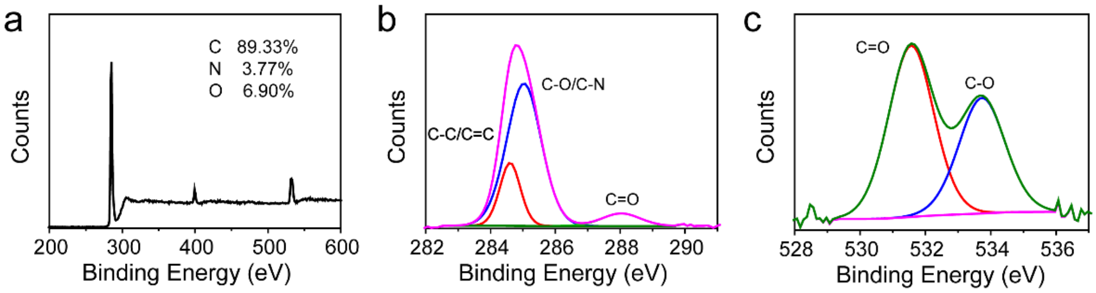

Fig. S1 a XPS spectrum of CDs. **b** High-resolution XPS spectra of C 1s and (**c**) O 1s.

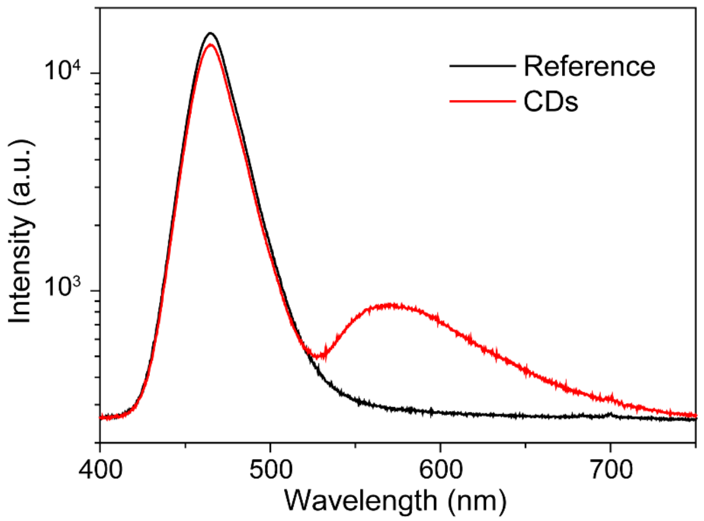

Fig. S2 Excitation line of reference and emission spectrum of CDs collected by an integration sphere spectroradiometer system.

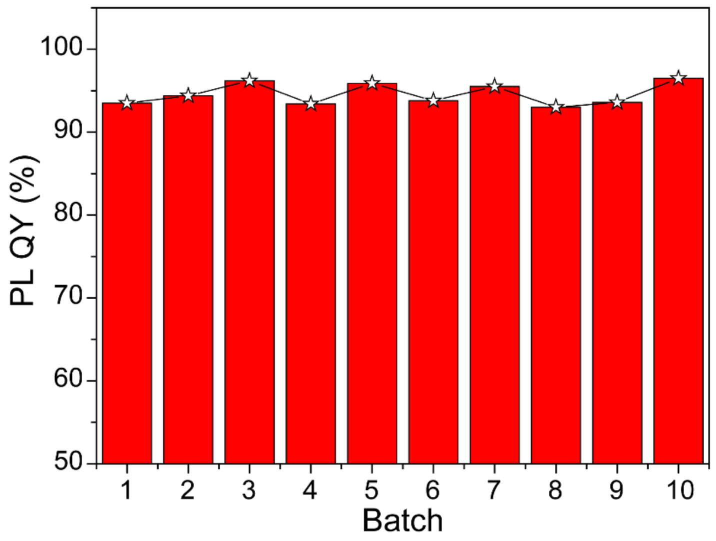

Fig. S3 PLQY results of ten batches of CDs.

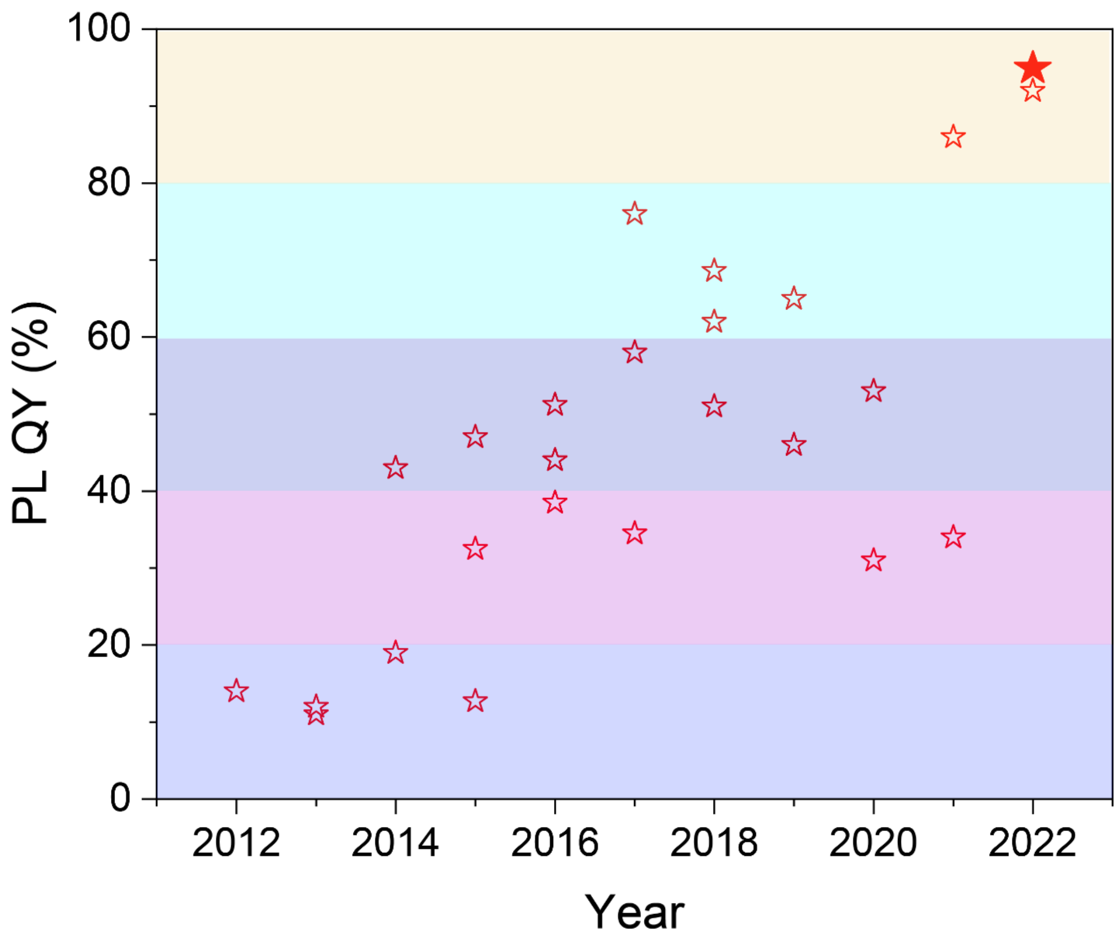

Fig. S4 Comparison of the PL QY of the yellow-emitting CDs. A detailed data point is presented in Table S1.

Note: In the work "Yellow-Emissive Carbon Dots with High Solid-State Photoluminescence" (e.g.10.1002/adfm.202110393), the authors reported heat treatment of citric acid and urea in toluene solvents, yielding CDs with PL QY of 92%. The high fluorescence of CDs came from the functionalized conjugated sp^2 carbon domains with edge groups (fused aromatic rings), and the luminescence position was affected by the size of the fused rings. However, the QY of the crude product is only 53%. Cumbersome column chromatography separation means must be used in order to obtain such an efficient yellow CDs, which is very detrimental to their mass production. In our work, CDs with near-unity emission efficiency have been prepared via atomic condensation of doped pyrrolic nitrogen, which can highly localize the excited states thus lead to the formation of bound excitons and the symmetry break of

the π–electron conjugation. These yellow-emitting CDs were be directly prepared with a heteroatom-doping solvothermal method with Methyl red as carbon sources and o-phenylenediamine as doping sources. The synthesis strategy is quite different, and the preparation method is relatively simple compared with the literature work.

As you mentioned about stability against UV irradiation for the CDs@PS film in our work, we have compared some previous reports with our results on the stability of CDs (Table S6). As shown in the table, the CDs@PS film has a considerable stability under a long-term UV irradiation with the duration of continuous irradiation exceeding 200 hours. Even so, 10% reduction of PL intensity for the CDs@PS film is detrimental and a hinder to their practical application in WLEDs. The low UV stability of CDs may originate from their surface functional groups or π conjugate system related fluorescence emission, which have a weak bond binding energy. Moreover, to improve the stability against UV irradiation, some protection and passivation measures can be used and confirmed by relevant reports. For instance, Wang *et al*. designed a novel method of physically embedding solid-state CDs into silicon matrix, which can effectively improve the stability of UV resistance (DOI: 10.1016/j.carbon.2017.10.04). Hence, this stability related issues can be addressed in our future work.

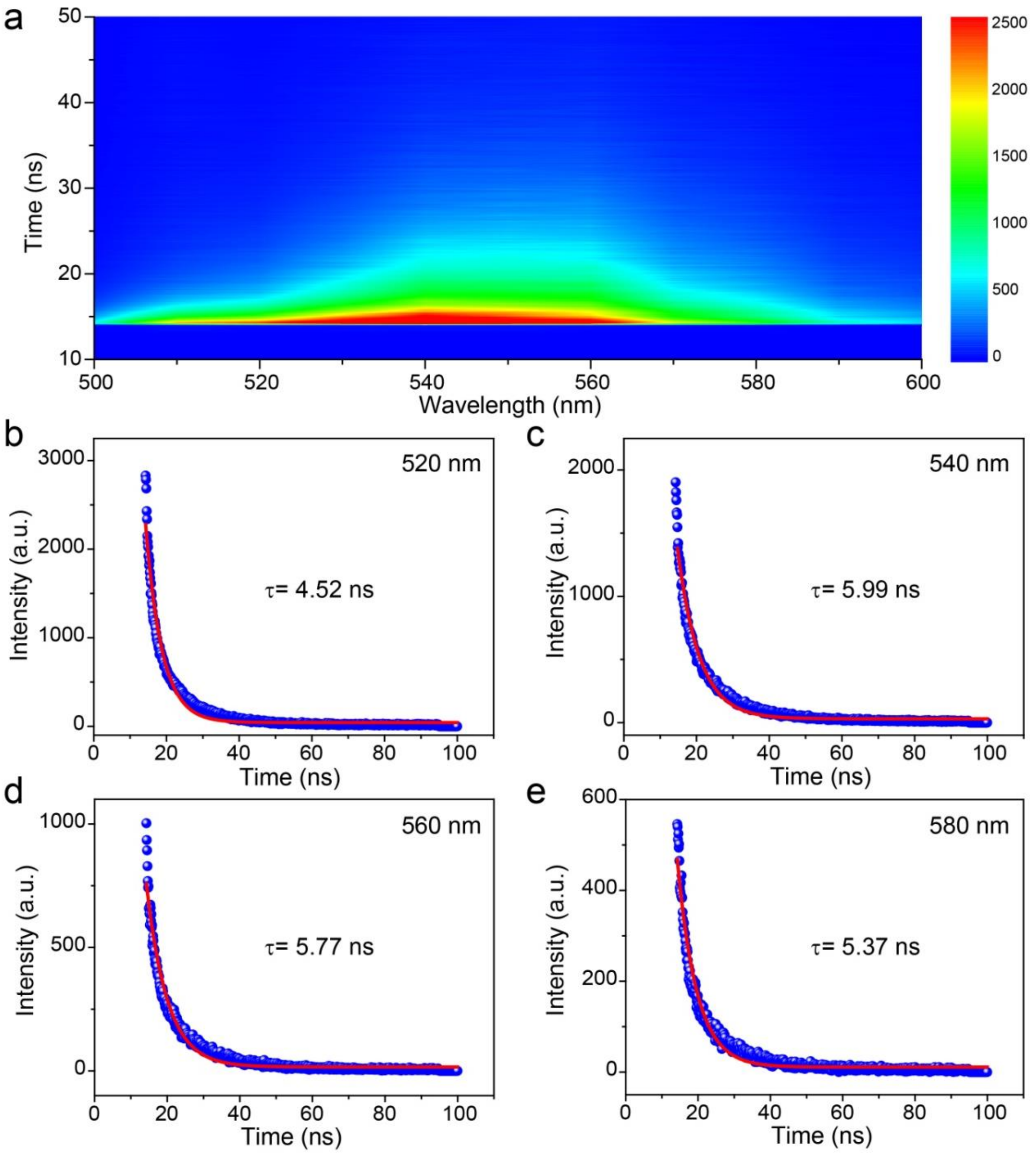

Fig. S5 a Time-resolved photoluminescence decay spectra of the CDs. **b** Single exponential fitting results of photoluminescence decay curves with wavelength of 520, (**c**) 540, (**d**) 560 and (**e**) 580 nm.

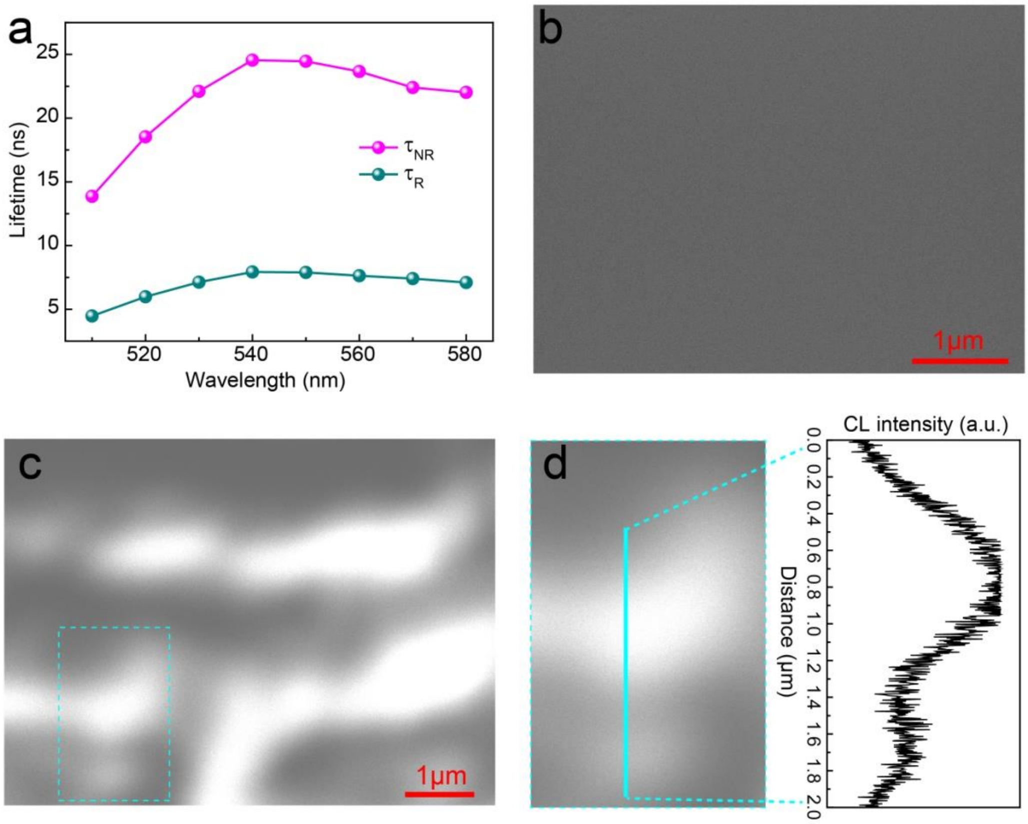

Fig. S6 a Values of τ_R and τ_{NR} for the emission at 300 K as functions of monitored different emission wavelengths in CDs. b Cathodoluminescence (CL) mapping measurement results of silicon substrate and (**c**) CD films. **d** Enlarged image of Fig. c with line distribution of the corresponding CL intensity.

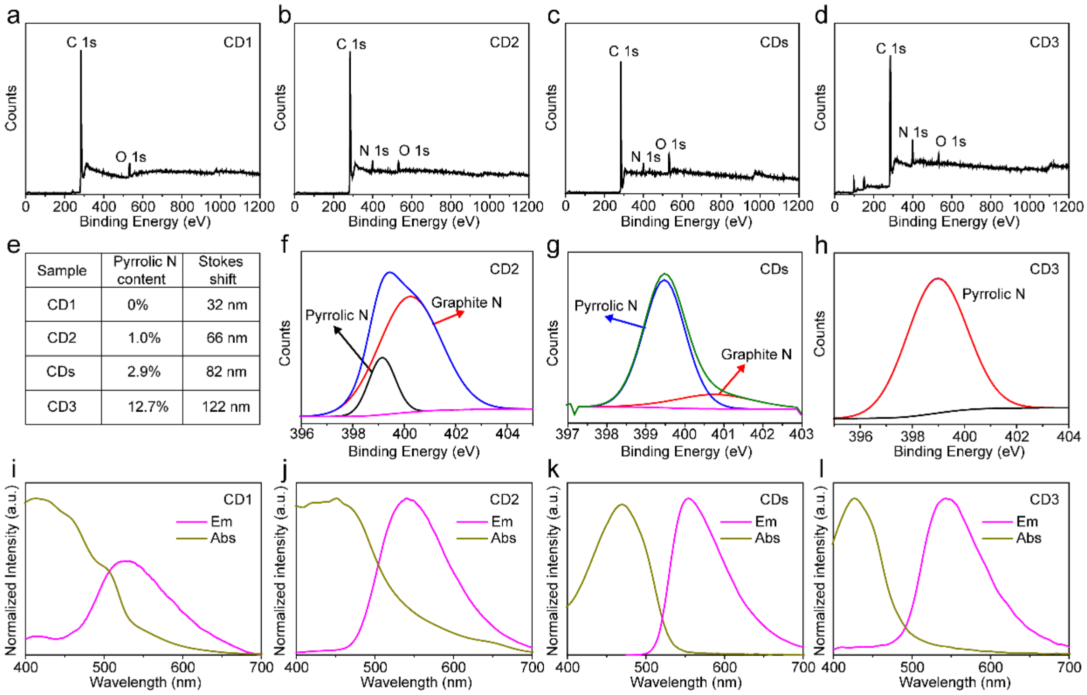

Sample	Pyrrolic N content	Stokes shift
CD1	0%	32 nm
CD2	1.0%	66 nm
CDs	2.9%	82 nm
CD3	12.7%	122 nm

Fig. S7 a XPS spectra of CD1, (**b**) CD2, (**c**) yellow-emitting CD (CDs) and (**d**) CD3. **e** Stokes shift results of these CDs. **f** High-resolution N 1s spectra of CD2, (**g**) CDs and (**h**) CD3. **i** PL spectra and absorption spectra of CD1, (**j**) CD2, (**k**) CDs and (**l**) CD3, respectively.

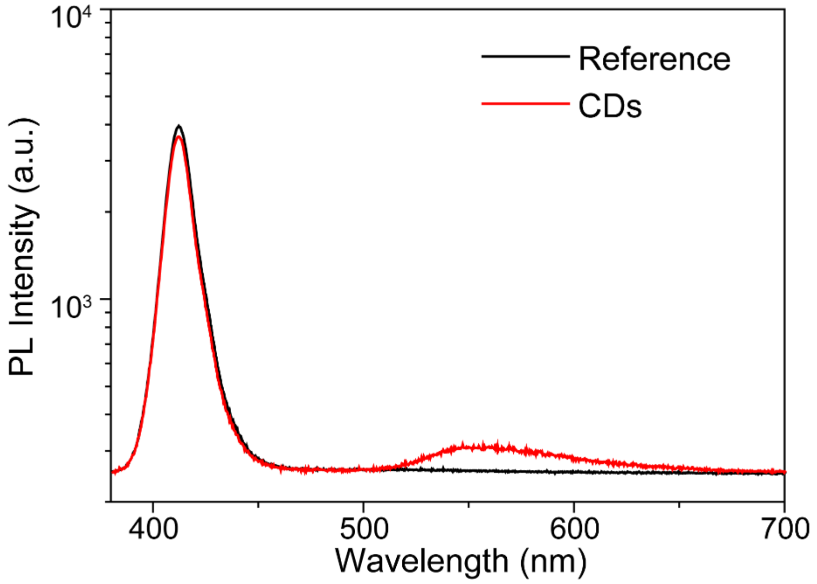

Fig. S8 Excitation line of reference and emission spectrum of CDs@PS composite film collected by an integration sphere spectroradiometer system.

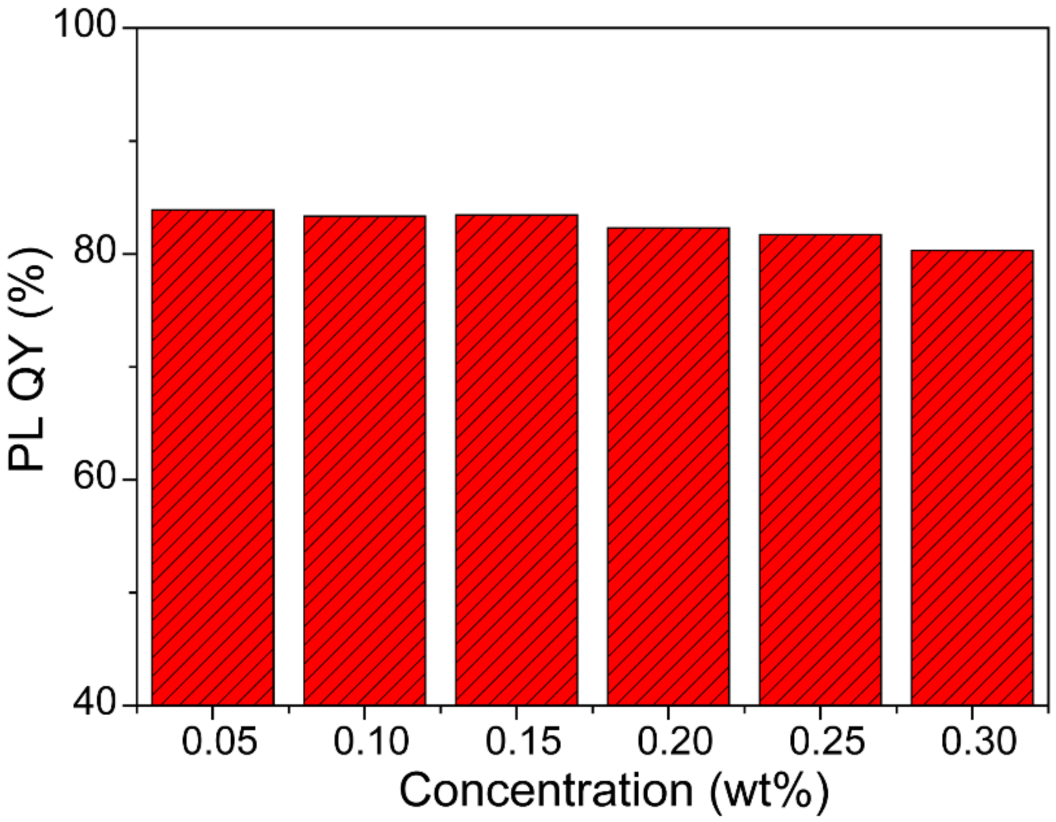

Fig. S9 PLQY of the yellow-emitting composite films with different CDs concentration.

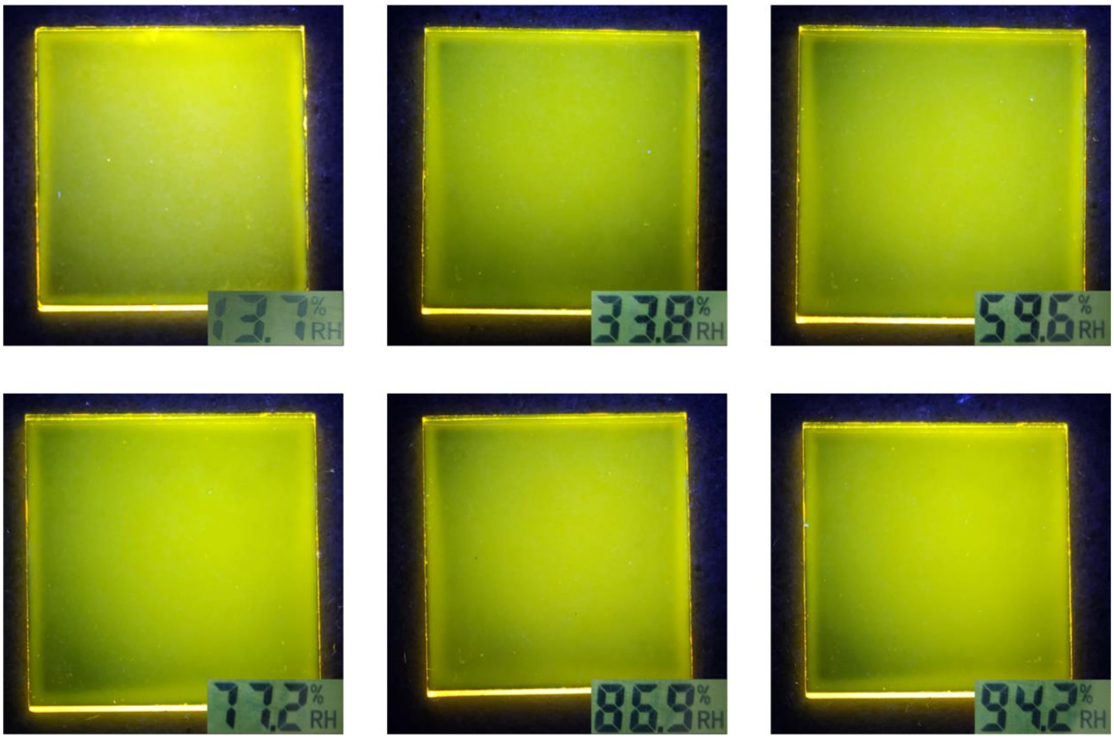

Fig. S10 Fluorescent images of the CD films in all kinds of humid environments with the humidity ranging from 13.7 to 94.2%.

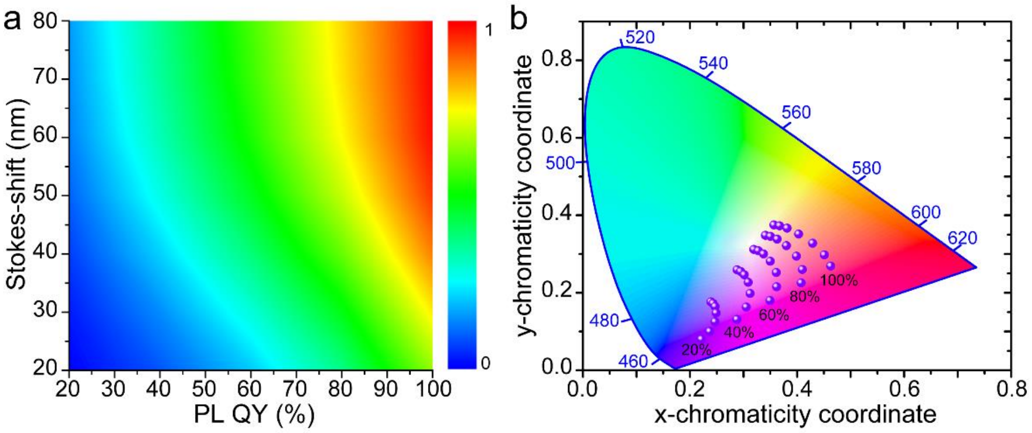

Fig. S11 Simulation results of the LE of a series of WLEDs with PLQY of 20, 40, 60, 80 and 100% (**a**) and Stokes shifts ranging from 20 nm to 80 nm (**b**).

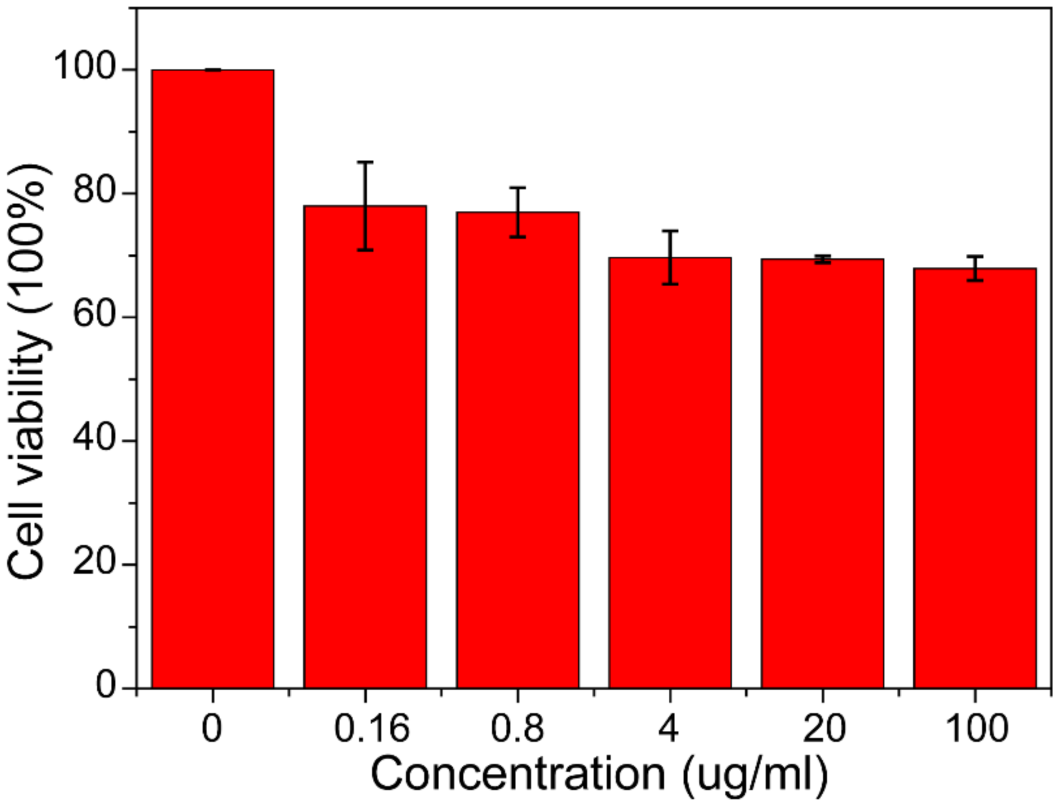

Fig. S12 Cell viability results of the CDs.

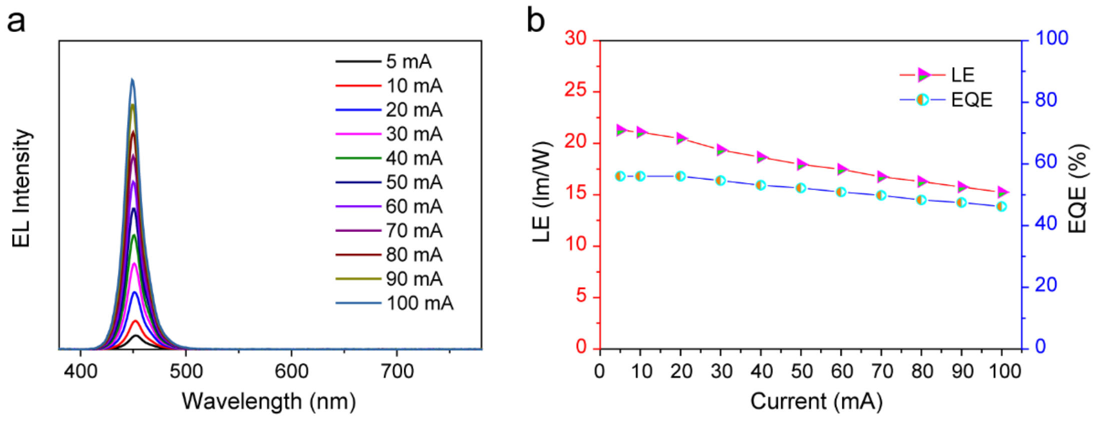

Fig. S13 a EL spectra, (**b**) luminous efficiency (LE) and external quantum efficiency (EQE) of the blue LED chip with drive current ranging from 5 to 100 mA.

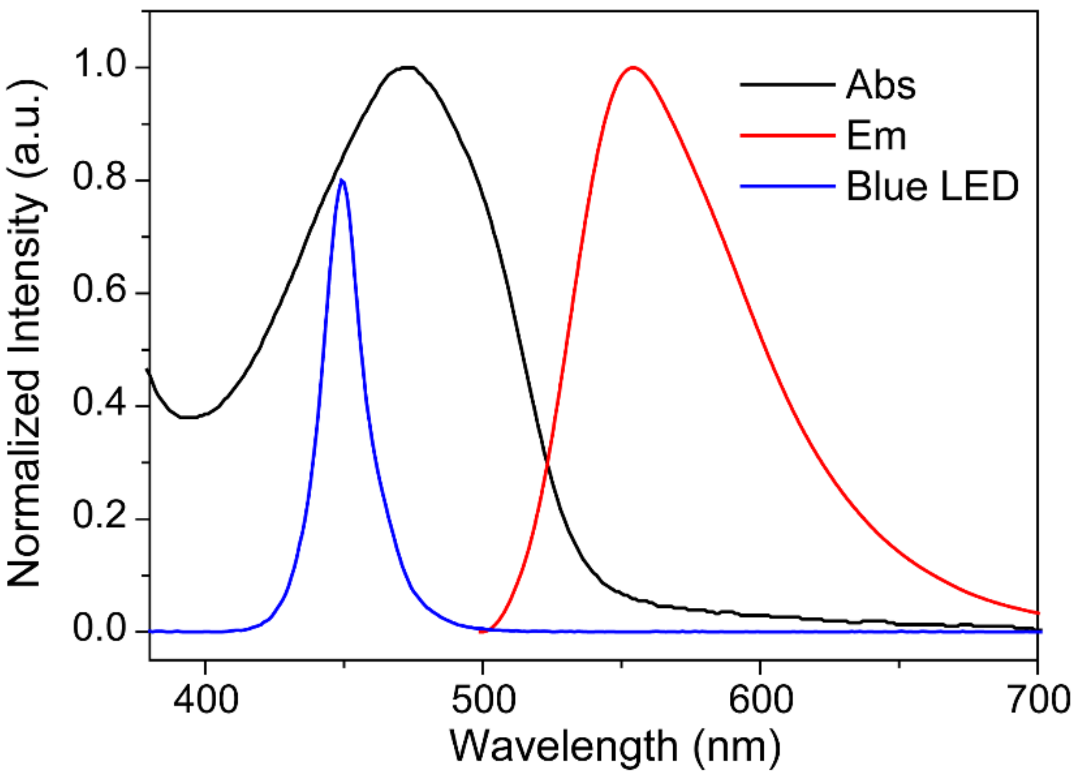

Fig. S14 PL and absorption spectra of CDs and EL spectrum of the blue LED chip.

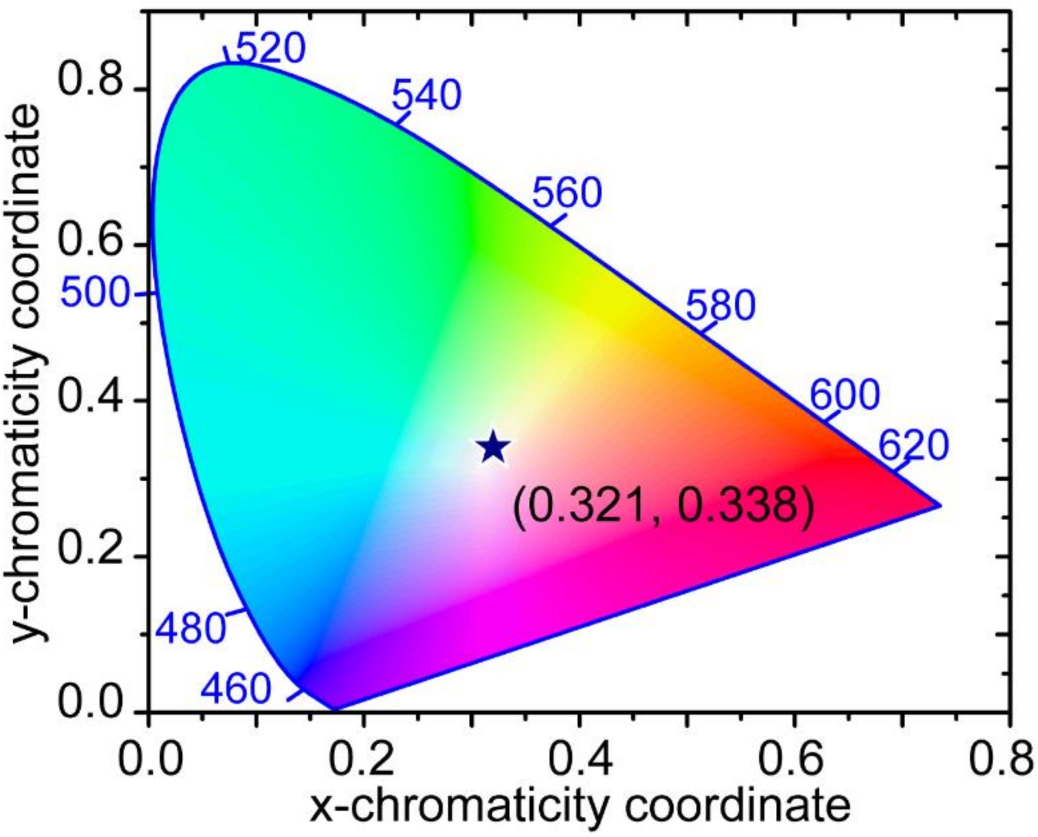

Fig. S15 Color coordinate of the CDs based WLED.

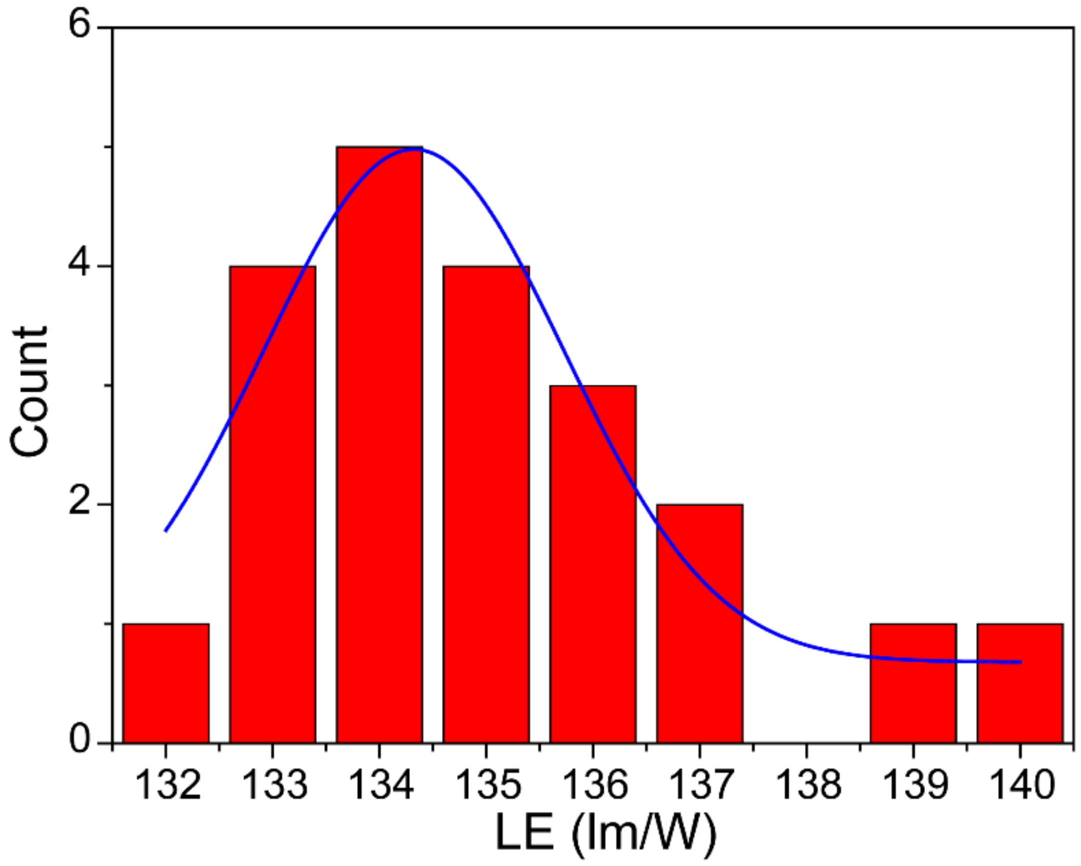

Fig. S16 Statistical results of LE of 20 WLEDs.

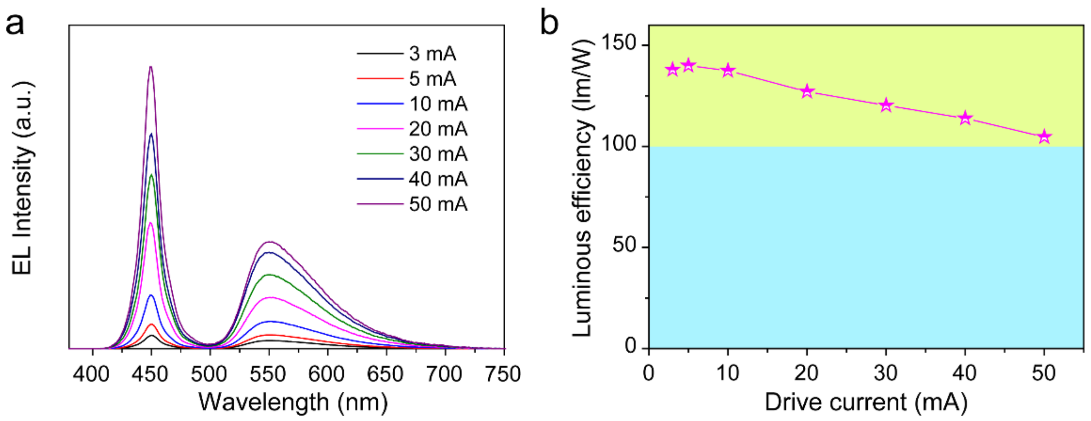

Fig. S17 a EL spectra and (**b**) luminous efficiency of the CD based WLEDs with drive current from 3 mA to 50 mA.

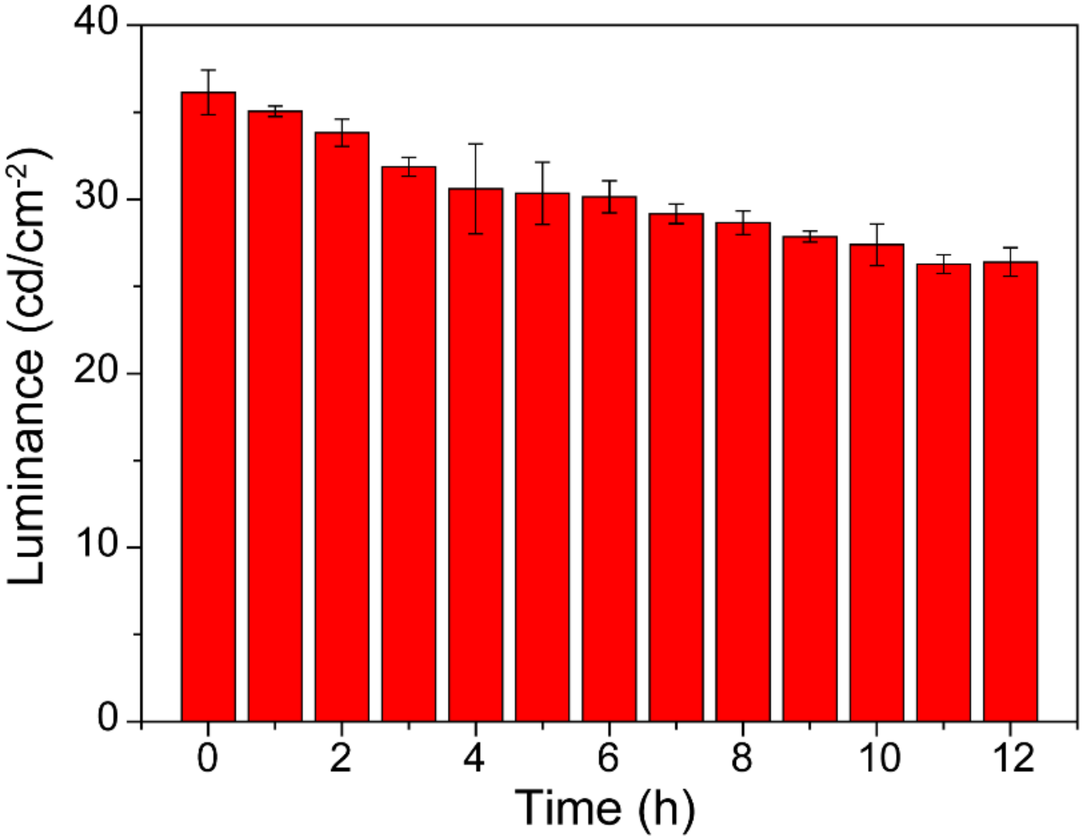

Fig. S18 Stability of the WLED with continuous operation.

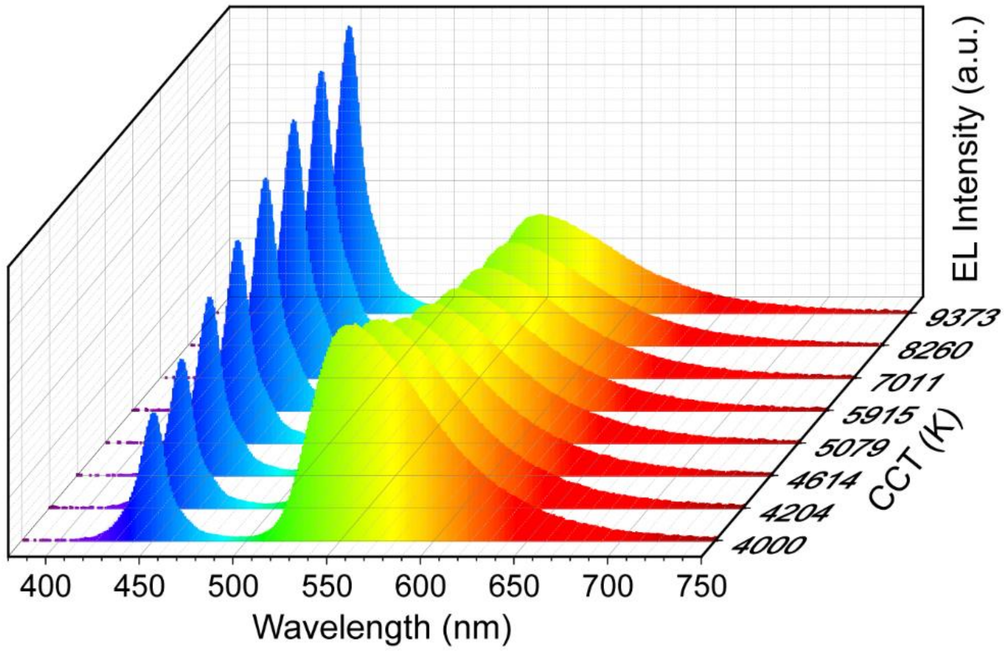

Fig. S19 EL spectra of the WLEDs with different CCT.

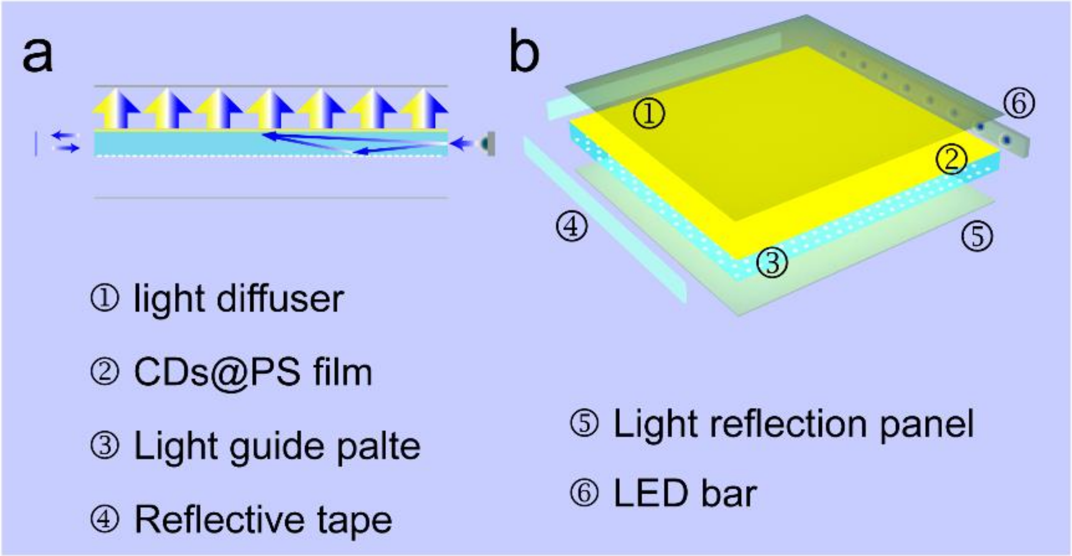

Fig. S20 Schematic diagram of the flat panel illumination system.

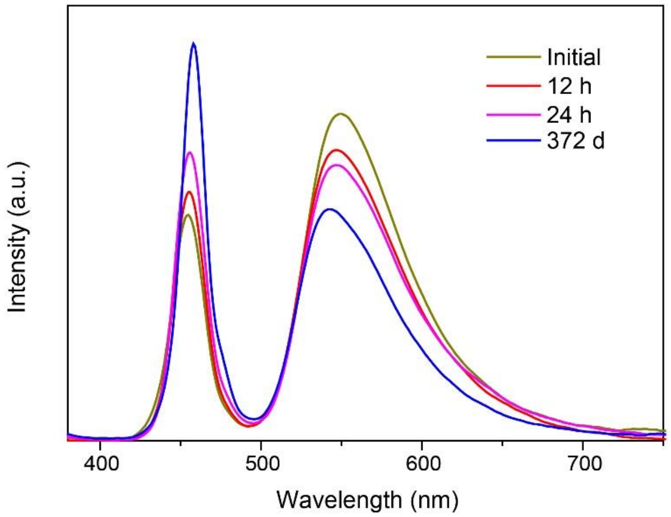

Fig. S21 EL spectra of the area light after operating12 hours (red line) and 24 hours (magenta line). The blue line is EL spectrum of the area light after placing in the air for 372 days.

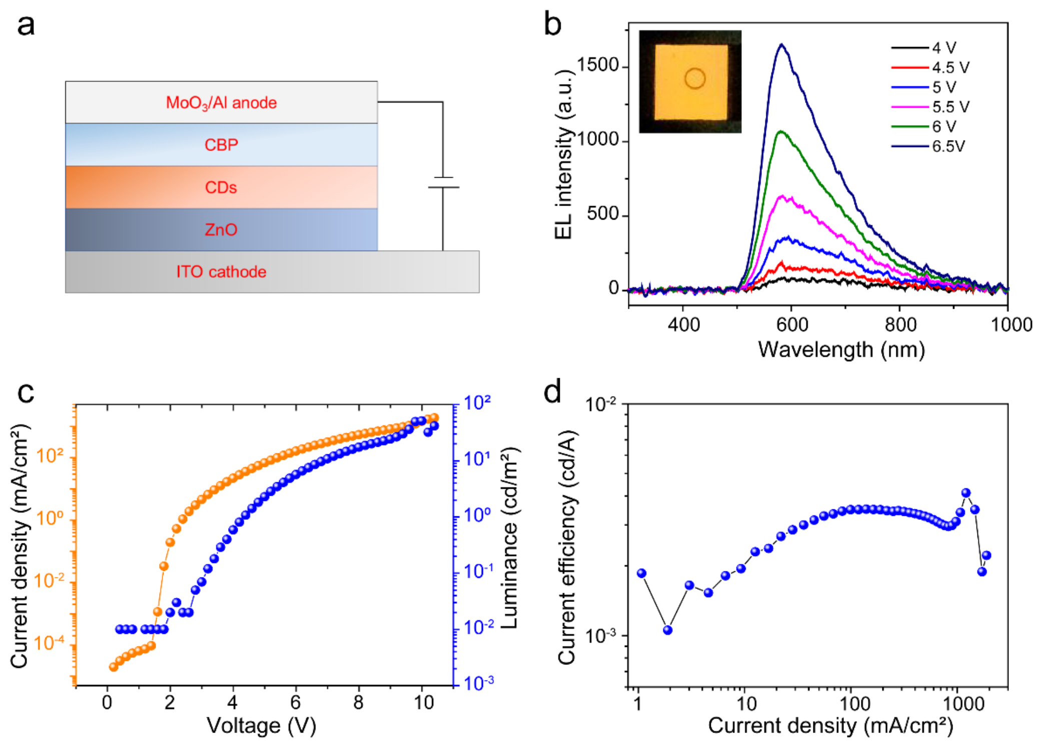

Fig. S22 (a) Structure of the CD-based electroluminescent device. (b) Electroluminescent spectra of the CD-based electroluminescent device at different voltage. The inset is the picture of the device. (c) Current density and luminance of the CD-based electroluminescent device at different voltage. (d) Current efficiency of the CD-based electroluminescent device with different current density.

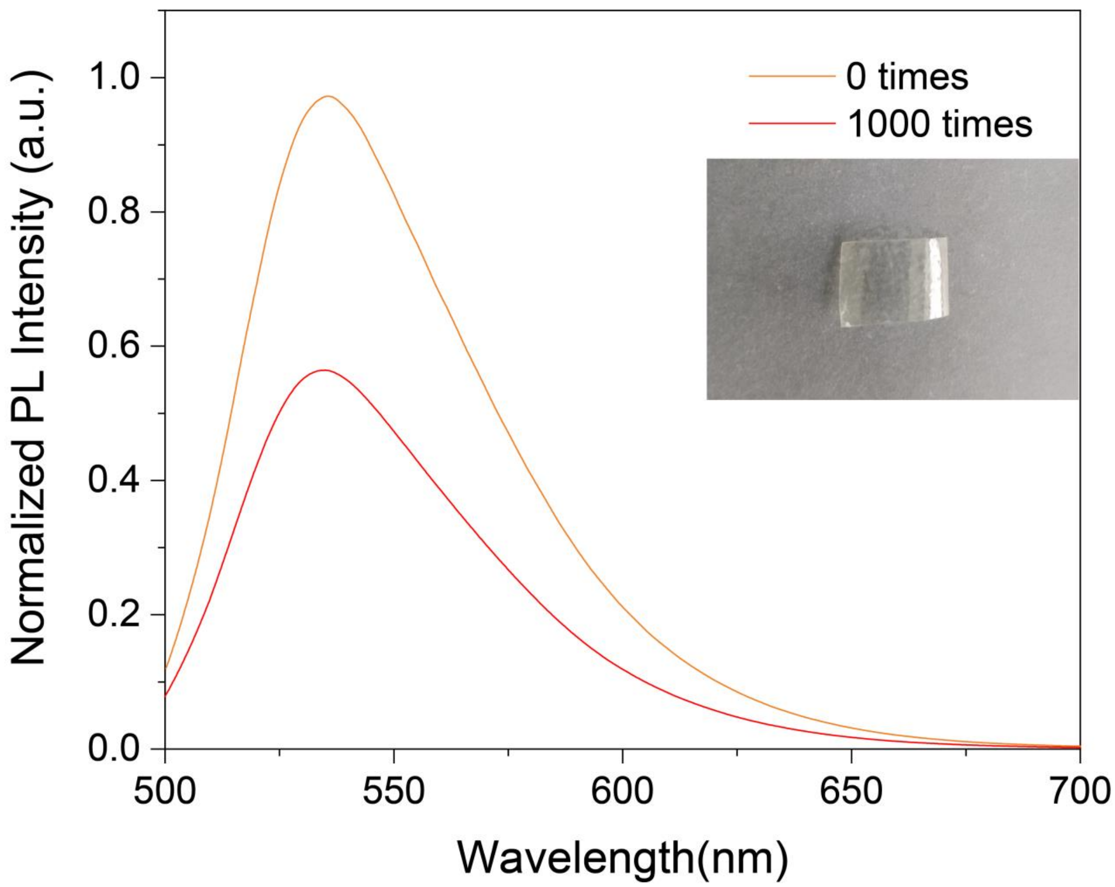

Fig. S23 Comparison of the fluorescence performance of the CD films after 1000-time bends. The inset is the photo of the film after 1000-time bends.

Note: We have carried out bending test on the composite film (Fig. S23). The CDs@PS composite films can be bent more than 1000 times without any breakage. After 1000-time bends, the composite film still maintained about 60% in luminous intensity, demonstrating good fluorescence emission stability.

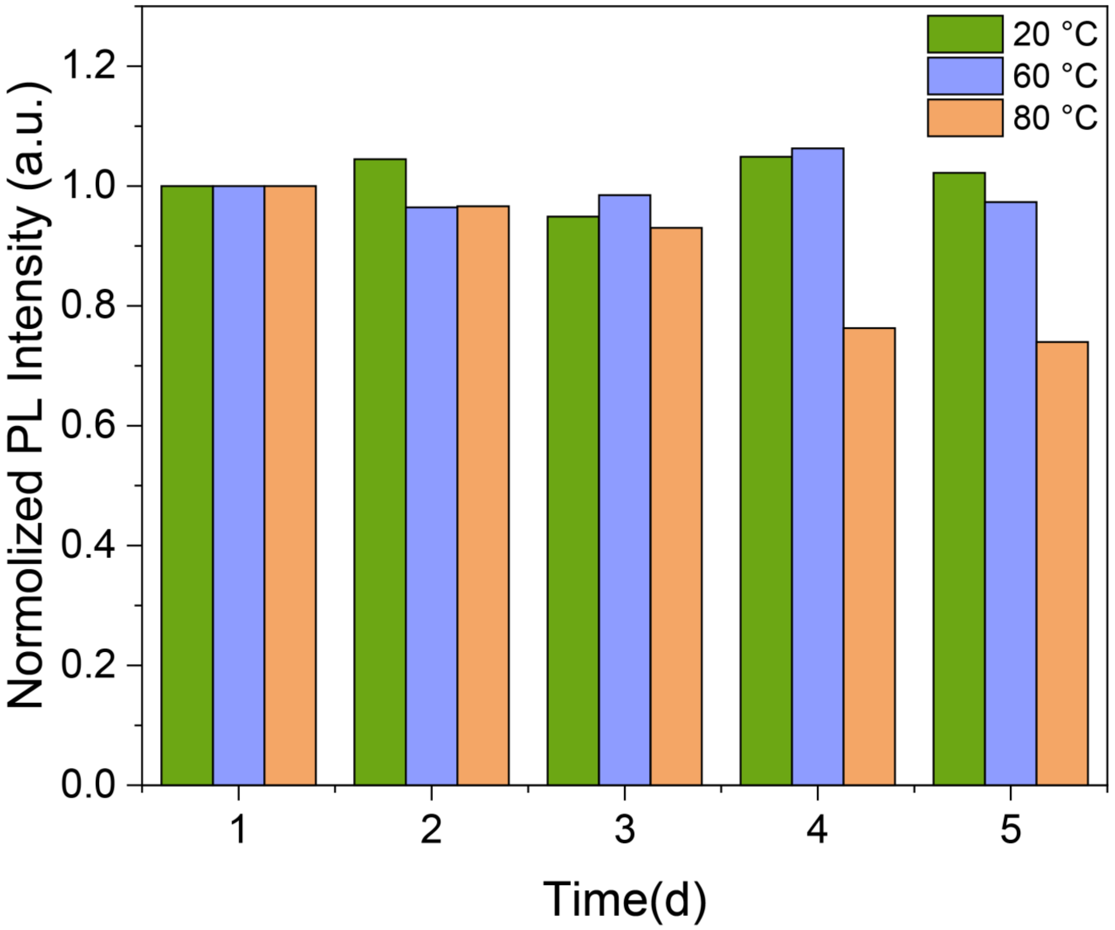

Fig. S24 Stability of the original CDs in dichloromethane solution under different temperature environments.

Note: We have tested the stability of the original CDs in dichloromethane solution under different temperature environments from 20 to 80 °C. As shown in Fig. S24, the CD solution still has good stability in variable temperature environment more than 120 hours, keeping larger than 70% of the original PL intensity even the solution temperature reaches to 80 °C.

TABLES

Table S1 PL QY of the reported yellow-emitting CDs.

YEAR	PLQY	REF.
2012	14	[8]
2013	11	[9]
2013	12	[10]
2014	19	[11]
2014	43	[12]
2015	12.7	[13]
2015	32.5	[14]
2015	47	[15]
2016	51.2	[16]
2016	38.5	[17]
2016	44	[18]
2017	58	[19]
2017	34.5	[20]
2017	76	[21]
2018	51	[22]
2018	68.6	[23]
2018	62	[24]

2019	46	[25]
2019	65	[26]
2020	31	[27]
2020	53	[28]
2021	86	[29]
2021	34	[30]
2022	92	[31]

Table S2 The lowest energy vertical electronic transitions of the nitrogen-doped systems NPx (x = 1, 2) and the nitrogen-free system P0, respectively.

System	S0→S1			S0→S2			S0→S3		
	E(eV)	λ(nm)	f(au)	E(eV)	λ(nm)	f(au)	E(eV)	λ(nm)	f(au)
P0	2.243	552.8	0	2.387	519.4	0	2.796	443.5	1.506
NP1	2.284	542.8	0.005	2.38	520.9	0.147	2.819	439.9	1.318
NP2	2.316	535.3	0.403	2.342	529.4	0.046	2.904	427.0	1.185

Table S3 Major MO involvement and CI contribution (%) for the electronic transitions into the first (S0→S1), second (S0→S2) and third (S0→S3) excited states in the nitrogen-doped systems NPx (x= 1-2) and the nitrogen-free system P0, respectively.

System	S0→S1		S0→S2		S0→S3	
	MO	CI (%)	MO	CI (%)	MO	CI (%)
P0	H-1→L	49.7	H→L	49.8	H→L+1	48.8
	H→L+1	49.7	H-1→L+1	49.7	H-1→L	48.8
NP1	H-1→L	53.7	H→L	77.3	H→L+1	52.1
	H→L+1	45.5	H-1→L+1	22	H-1→L	44.3
NP2	H→L	91.5	H-1→L	64.4	H→L+1	62.9
	H-1→L+1	7.9	H→L+1	34.9	H-1→L	33.9

Table S4 Calculated S1→S0, S2→S0 and S3→S0 fluorescence for the nitrogen-doped systems NPx (x= 1-2) and the nitrogen-free system P0, respectively.

System	S1→S0			S2→S0			S3→S0		
	E(eV)	λ(nm)	f(au)	E(eV)	λ(nm)	f(au)	E(eV)	λ(nm)	f(au)
P0	2.170	571.5	0	2.320	534.5	0	2.736	453.2	1.520
NP1	2.203	562.8	0.004	2.306	537.6	0.143	2.751	450.7	1.336
NP2	2.150	576.7	0.516	2.273	545.5	0.055	2.847	435.5	1.175

Table S5 Luminous efficiencies of white LEDs based on CDs.

YEAR	LE (lm/W)	REF.
2015	4.9	[32]
2016	20	[18]
2017	67	[33]
2017	8.34	[34]
2017	31.3	[35]
2017	36.7	[36]
2018	8.8	[37]
2018	44.15	[38]
2018	71.75	[39]
2019	86.5	[40]
2019	30.54	[41]
2020	51.3	[42]

Table S6 Comparison of the fluorescence stability of the CDs under ultraviolet light.

Reports	UV radiation time	Ratio of residual fluorescence intensity	DOI
1	1 h	100% (1.0→1.0)	10.1016/j.carbon.2017.10.04
2	4 h	55.6% (0.9→0.5)	10.1016/j.carbon.2022.07.034.
3	5 h	88% (96%→84.5%)	10.3390/ma15072395
4	8 h	95%（1.0→0.95）	10.1016/j.jmst.2020.03.020
5	8 h	100% (4.4×10E6→4.4×10E6)	10.1016/j.snb.2018.03.183
6	18 h	35% (1.0→0.35)	10.1039/c7nr03648e
7	100 h	96.4% (100%→96.4%)	10.1007/s40843-022-2009-y
**8**	**240 h**	**90% (1.0→0.9)**	**Our work**

References

[1] Luo, J., Wang, X., Li, S. *et al*. Efficient and stable emission of warm-white light from lead-free halide double perovskites. *Nature* **563**, 541-545 (2018).

[2] Chichibu, S. F., Uedono, A., Onuma, T. *et al*. Origin of defect-insensitive emission probability in In-containing (Al,In,Ga)N alloy semiconductors. *Nat. Mater.* **5**, 810-816 (2006).

[3] Becke, A. D. Density-Functional Thermochemistry. III. The Role of Exact Exchange. *J. Chem. Phys*. **98**, 5648-5652 (1993).

[4] Grimme, S., Antony, J., Ehrlich, S., Krieg, H. A Consistent and Accurate Ab Initio Parametrization of Density Functional Dispersion Correction ((DFT-D) for the 94 Elements H-Pu. *J. Chem. Phys*. **132,** 154104 (2010).

[5] Rassolov, V. A., Ratner, M. A., Pople, J. A., *et al*. 6-31G* Basis Set for Third-Row Atoms. *J. Comput. Chem*. **22,** 9976–9984 (2001).

[6] Frisch, M. J., Trucks, G. W., Schlegel, H. B. *et al.* Gaussian 09, Revision D.01; Gaussian, Inc.: Wallingford, CT, (2009).

[7] Sadeghi, S., Kumar, B. G., Melikov, R. *et al*. Quantum dot white LEDs with high luminous efficiency. *Optica* **5**, 793-802 (2018).

[8] Zhang, M. *et al*. Facile synthesis of water-soluble, highly fluorescent graphene quantum dots as a robust biological label for stem cells. *J. Mater. Chem.* **22**, 7461-7467 (2012).

[9] Fu, X., Li, D. & Zhang, Y. Synthesis of photoluminescent carbon nanoparticles from graphite. *J. Nanopart. Res.* **15**, 1598 (2013).

[10] Bhunia, S., Saha, A., Maity, A. *et al*. Carbon Nanoparticle-based Fluorescent

Bioimaging Probes. Sci. Rep. **3**, 1473 (2013).

[11] Yang, S., Sun, J., Li, X. *et al*. Large-scale fabrication of heavy doped carbon quantum dots with tunable-photoluminescence and sensitive fluorescence detection. *J. Mater. Chem. A* **2**, 8660-8667 (2014).

[12] Yan, S., Yang, S., He, L. *et al*. Quantum size effect of poly(o-phenylenediamine) quantum dots: From controllable fabrication to tunable photoluminescence properties. *Synthetic Met.* **198**, 142-149 (2014).

[13] Ding, H., Yu, S. B., Wei, J. S. *et al*. Full-color light-emitting carbon dots with a surface-state-controlled luminescence mechanism. *ACS Nano* **10**, 484-491 (2016).

[14] Jiang, K., Sun, S., Zhang, L. *et al*. Bright-yellow-emissive N-doped carbon dots: Preparation, cellular imaging, and bifunctional sensing. *ACS Appl. Mater. Interfaces* **7**, 23231-23238 (2015).

[15] Sun, C., Zhang, Y., Kalytchuk, S. *et al*. Down-conversion monochromatic light-emitting diodes with the color determined by the active layer thickness and concentration of carbon dots. *J. Mater. Chem. C* **3**, 6613-6615 (2015).

[16] Cheng, J., Wang, C. F., Zhang, Y. *et al*. Zinc ion-doped carbon dots with strong yellow photoluminescence. *RSC Adv*. **6**, 37189-37194 (2016).

[17] Song, L., Cui, Y., Zhang, C. *et al*. Microwave-assisted facile synthesis of yellow fluorescent carbon dots from o-phenylenediamine for cell imaging and sensitive detection of $Fe3^{+}$ and H_2O_2. *RSC Adv.* **6**, 17704-17712 (2016).

[18] Kim, T. H., White, A. R., Sirdaarta, J. P. *et al*. Yellow-emitting carbon nanodots and their flexible and transparent films for white LEDs. *ACS Appl. Mater. Interfaces* **8**,

33102-33111 (2016).

[19] Yuan, F., Wang, Z., Li, X. *et al*. Bright multicolor bandgap fluorescent carbon quantum dots for electroluminescent light-emitting diodes. *Adv. Mater*. **29**, 1604436 (2017).

[20] Wang, H., Sun, C., Chen, X. *et al*. Excitation wavelength independent visible color emission of carbon dots. *Nanoscale* **9**, 1909-1915 (2017).

[21] Wang, T. Y., Chen, C. Y., Wang, C. M. *et al*. Multicolor functional carbon dots via one-step refluxing synthesis. *ACS Sens.* **2**, 354-363 (2017).

[22] Ding, H., Wei, J. S., Zhang, P. *et al*. Solvent-controlled synthesis of highly luminescent carbon dots with a wide color gamut and narrowed emission peak widths. *Small* **14**, e1800612 (2018).

[23] Liu, Y., Chao, D., Zhou, L. *et al*. Yellow emissive carbon dots with quantum yield up to 68.6% from manganese ions. *Carbon* **135**, 253-259 (2018).

[24] Yuan, F., Yuan, T., Sui, L. *et al*. Engineering triangular carbon quantum dots with unprecedented narrow bandwidth emission for multicolored LEDs. *Nat. Commun.* **9**, 2249 (2018).

[25] Han, Z., Ni, Y., Ren, J. *et al*. Highly efficient and ultra-narrow bandwidth orange emissive carbon dots for microcavity lasers. *Nanoscale* **11**, 11577-11583 (2019).

[26] Zhang, Y., Zhuo, P., Yin, H. *et al*. Solid-State Fluorescent Carbon Dots with Aggregation-Induced Yellow Emission for White Light-Emitting Diodes with High Luminous Efficiencies. *ACS Appl. Mater. Interfaces* **11**, 24395-24403 (2019).

[27] Zhang, S., Ji, X., Liu, J. *et al*. One-step synthesis of yellow-emissive carbon dots

with a large Stokes shift and their application in fluorimetric imaging of intracellular pH. *Spectrochim. Acta A* **227**, 117677 (2020).

[28] Zheng, K., Li, X., Chen, M. *et al*. Controllable synthesis highly efficient red, yellow and blue carbon nanodots for photo-luminescent light-emitting devices. *Chem. Eng. J.* **380**, 122503 (2020).

[29] Li, J., Zhao, H., Zhao, X., *et al*. Red and yellow emissive carbon dots integrated tandem luminescent solar concentrators with significantly improved efficiency. *Nanoscale*. **13**, 9561 (2021).

[30] Kainth, S., Sharma, V., Bhagat, M. *et al*. Yellow emissive carbon dots in ludox silica matrix with anticancer activity for enhanced imaging of developed sweat latent fingermarks. *Mater. Today Chem.* **23**, 100659 (2022).

[31] Guo, J., Lu, Y., Xie, A., et al. Yellow-Emissive carbon dots with high solid-state photoluminescence. *Adv. Funct. Mater.* **32**, 2110393 (2022).

[32] Sun, C., Zhang, Y., Sun, K. *et al*. Combination of carbon dot and polymer dot phosphors for white light-emitting diodes. *Nanoscale* **7**, 12045-12050 (2015).

[33] Chen, D., Gao, H., Chen, X. *et al*. Excitation-independent dual-color carbon dots: Surface-state controlling and solid-state lighting. *ACS Photonics* **4**, 2352-2358 (2017).

[34] Tian, Z., Zhang, X., Li, D. *et al*. Full-color inorganic carbon dot phosphors for white-light-emitting diodes. *Adv. Optical Mater.* **5**, 1700416 (2017).

[35] Wang, Z., Yuan, F., Li, X. *et al*. 53% Efficient red emissive carbon quantum dots for high color rendering and stable warm white-light-emitting diodes. *Adv. Mater.* **29**, 1702910 (2017).

[36] Zhu, J., Bai, X., Zhai, Y. *et al*. Carbon dots with efficient solid-state photoluminescence towards white light-emitting diodes. *J. Mater. Chem. C* **5**, 11416-11420 (2017).

[37] Yuan, K., Zhang, X., Qin, R. *et al*. Surface state modulation of red emitting carbon dots for white light-emitting diodes. *J. Mater. Chem. C* **6**, 12631-12637 (2018).

[38] Han, Z., Wang, K., Du, F., *et al*. High efficiency red emission carbon dots based on phenylene diisocyanate for trichromatic white and red LEDs. *J. Mater. Chem. C* **6**, 9631-9635 (2018).

[39] Yuan, B., Guan, S., Sun, X. *et al*. Highly efficient carbon dots with reversibly switchable green-red emissions for trichromatic white light-emitting diodes. *ACS Appl. Mater. Interfaces*, **10**, 16005-16014 (2018).

[40] Yuan, F., He, P., Xi, Z. *et al*. Highly efficient and stable white LEDs based on pure red narrow bandwidth emission triangular carbon quantum dots for wide-color gamut backlight displays. *Nano Res.* **12**, 1669-1674 (2019).

[41] Cao, M., Xia, C., Xia, J. *et al*. A yellow carbon dots-based phosphor with high efficiency for white light-emitting devices. *J. Lumin.* **206**, 97-104 (2019).

[42] Wei, J. Y., Lou, Q., Zang, J. H. *et al*. Scalable synthesis of green fluorescent carbon dot powders with unprecedented efficiency. *Adv. Optical Mater.* **8**, 1901938 (2020).
